# Monolithic 3D integration of 2D transistors and vertical RRAMs in 1T–4R structure for high-density memory

**DOI:** 10.1038/s41467-023-41736-2

**Published:** 2023-09-23

**Authors:** Maosong Xie, Yueyang Jia, Chen Nie, Zuheng Liu, Alvin Tang, Shiquan Fan, Xiaoyao Liang, Li Jiang, Zhezhi He, Rui Yang

**Affiliations:** 1 University of Michigan—Shanghai Jiao Tong University Joint Institute, Shanghai Jiao Tong University, Shanghai, China; 2 School of Electronic Information and Electrical Engineering, Shanghai Jiao Tong University, Shanghai, China; 3 Department of Electrical Engineering, Stanford University, Stanford, California USA; 4 School of Microelectronics, Xi’an Jiaotong University, Xi’an, Shaanxi China; 5 MoE Key Lab of Artificial Intelligence, Shanghai Jiao Tong University, Shanghai, China; 6 Shanghai Qi Zhi Institute, Shanghai, China; 7 State Key Laboratory of Radio Frequency Heterogeneous Integration, Shanghai Jiao Tong University, Shanghai, China

**Keywords:** Electronic devices, Electrical and electronic engineering

## Abstract

Emerging data-intensive computation has driven the advanced packaging and vertical stacking of integrated circuits, for minimized latency and energy consumption. Yet a monolithic three-dimensional (3D) integrated structure with interleaved logic and high-density memory layers has been difficult to achieve due to challenges in managing the thermal budget. Here we experimentally demonstrate a monolithic 3D integration of atomically-thin molybdenum disulfide (MoS_2_) transistors and 3D vertical resistive random-access memories (VRRAMs), with the MoS_2_ transistors stacked between the bottom-plane and top-plane VRRAMs. The whole fabrication process is integration-friendly (below 300 °C), and the measurement results confirm that the top-plane fabrication does not affect the bottom-plane devices. The MoS_2_ transistor can drive each layer of VRRAM into four resistance states. Circuit-level modeling of the monolithic 3D structure demonstrates smaller area, faster data transfer, and lower energy consumption than a planar memory. Such platform holds a high potential for energy-efficient 3D on-chip memory systems.

## Abstract (web summary)

Designing a monolithic 3D structure with interleaved logic and high-density memory layers has been difficult to achieve due to challenges in managing the thermal budget. Here, the authors demonstrate a 3D integration of monolayer MoS_2_ transistors with 3D vertical RRAMs through a low-temperature fabrication process whose 1T–*n*R structure shows high promise for low-power and high-density memory applications.

## Introduction

With the development of data-intensive computation such as cloud computing, artificial intelligence, and edge computing, there is an increasing demand for bringing the high-density on-chip memory close to the computing units for efficient memory access and high computing performance^1^. The lateral dimension scaling of complementary–metal–oxide–semiconductor (CMOS) devices has been slowing down, which limits the on-chip memory capacity, while off-chip dynamic random-access memory (DRAM) usually incurs long latency and high energy consumption. Advanced packaging technologies using 2.5-dimensional (2.5D) silicon interposers and three-dimensional (3D) die stacking have been actively explored for higher integration density in the package. However, such integration is dependent on the costly through-silicon vias (TSVs), which usually have large pitch, poor yield, and low reliability^2^.

Monolithic 3D system features fine-grained integration of logic and memory layers, interconnected by dense nanoscale inter-layer vias (ILVs). This can result in up to 1000-fold reduction of energy–delay product in data-intensive applications due to the reduced latency and energy in memory access^3^. Such a monolithic 3D integration system requires the top-plane devices to be fabricated at low enough temperature (typically <400 °C)^4^ to ensure a small thermal budget and avoid affecting the devices underneath^5^. Prototype monolithic 3D structures have been achieved based on CMOS over CMOS CoolCube^TM^ system^6^, monolithically integrated metal-oxide-based thin-film transistors for embedded DRAMs^5^, indium gallium zinc oxide (IGZO) transistors integrated with resistive random-access memories (RRAMs)^7^, as well as carbon nanotube transistors integrated with RRAMs^4,8^. Yet the logic and high-density memory materials and devices that are suitable for monolithic 3D integration are still not fully optimized, and require careful selection and active exploration.

Transistors made of two-dimensional (2D) semiconductors have a naturally passivated surface to reduce the surface roughness scattering, and have an atomically thin channel for enhanced gate control and suppression of short-channel effects, which make them highly promising for scaled logic devices below the 3-nm technology node^9–12^. In addition, although the growth of 2D materials is usually performed at elevated temperatures, the low transfer temperature (usually below 150 °C) makes 2D materials particularly suitable for vertical heterogeneous integration^13^. Molybdenum disulfide (MoS_2_) as a 2D semiconductor with a bandgap of ~2 eV for monolayer, has enabled field-effect transistors (FETs) with 1-nm gate length^14,15^, shown improved contact^16,17^, and been integrated in a large scale with high yield and uniformity^18–20^. Microprocessors, memory, and in-memory computing based on MoS_2_ have also been demonstrated recently^21–26^. Therefore, MoS_2_ FETs are suitable as logic circuits or selecting devices for memory in monolithic 3D integrated systems.

Besides logic devices, the choice of high-density memory devices is also essential for monolithic 3D integrated systems. Metal-oxide RRAMs are non-volatile, scalable to ultra-small device sizes (<5 nm), suitable for multi-level storage, and are compatible with CMOS back–end–of–line processes^27–29^. Based on such emerging nonvolatile memory, storage-class memory and in-memory computing systems have been demonstrated^30–33^. 2D materials are also suitable as the switching layer of resistive memories, with hexagonal boron nitride (h-BN) and MoS_2_ RRAMs demonstrated in wafer scale^34,35^. In addition, 3D vertical RRAM (VRRAM) structures can further increase the memory density, which make them highly promising towards large-scale on-chip memory, physically unclonable functions, and in-memory computing^36–45^. 2D graphene has been used as the edge electrode in 3D VRRAMs^46^, and 2D transistors have been integrated with RRAMs or CMOS devices^47–51^. However, 2D transistors have not been integrated with 3D VRRAMs towards monolithic 3D integrated systems with high memory density.

In this work, we demonstrate the monolithic 3D integration of 2D MoS_2_ FETs and HfO_x_-based 3D VRRAMs, forming a one–transistor–four–VRRAMs (1T–4R) structure, with high density of memory layers (up to 4 layers) per unit area, and high density of total stacked device layers (up to 5 device layers). Electrical characterizations show high uniformity among the four layers of VRRAMs, and confirm that the bottom-plane device functionality is maintained after top-plane device fabrication, thanks to the low fabrication temperature during the whole process (≤300 °C), which allows further integration of more device layers vertically. The 2D MoS_2_ is grown by chemical vapor deposition (CVD) to enable uniform large-scale transistors, and the MoS_2_ FETs drive and select the VRRAM cells for multiple switching cycles. We have measured 40 VRRAMs in each of the four layers on the same chip, which demonstrates the potential for large-scale integration. Each layer of VRRAM can store up to eight stable resistance states, where the memory density is further boosted by combining with our vertically stacked four VRRAM layers. We have also performed parallel set and reset measurements and simulations on VRRAMs driven by the same MoS_2_ transistor to show the parallel programming capability of 3D VRRAM arrays. Circuit-level simulations show that compared with planar one–transistor–one–RRAM (1T–1R) configuration, such one–transistor–*n*–VRRAMs (1T–*n*R) 3D structure results in the reduction of memory area, read latency, and read energy consumption by up to 87.3%, 70.6% and 72.8%, respectively. Such structure is promising for continued vertical scaling towards an energy-efficient and high-density monolithic 3D integrated system.

## Results

### Monolithic 3D device structure

**Fig. 1:**
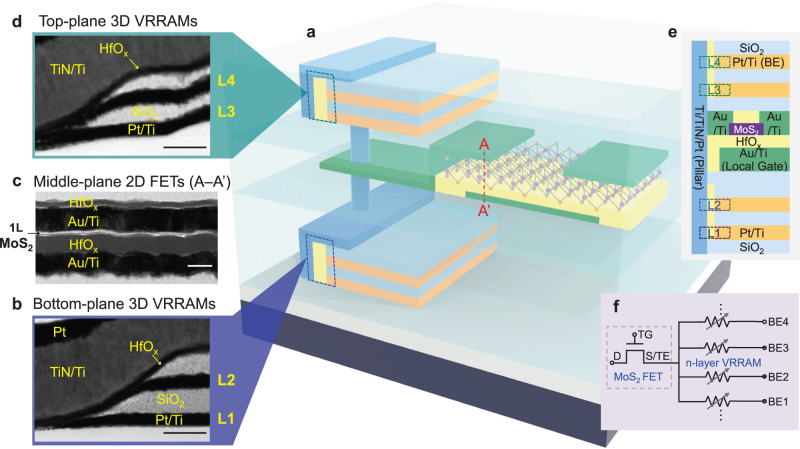
Monolithic 3D integration of 2D MoS_2_ transistors and 3D VRRAMs into a 1T–4R structure. **a** 3D schematic illustration of the 1T–4R structure. Cross-sectional TEM images of **b** the bottom-plane 3D VRRAMs (L1 and L2), **c** the middle-plane 2D MoS_2_ FETs as noted by the A–A’ dashed line in **a**, and **d** the top-plane 3D VRRAMs (L3 and L4). *Scale bars*: 50 nm. **e** Cross-sectional illustration of the structure, showing the materials, as well as the top electrode (TE) and the bottom electrode (BE). **f** Equivalent circuit of the 1T–*n*R structure.

To demonstrate the fine-grained integration of logic and memory layers, we use HfO_x_-based 3D VRRAMs as the high-density memory, which are selected and driven by the monolayer MoS_2_ transistors (Fig. 1a). We first design and fabricate the two-layer 3D VRRAMs on the bottom plane (see Methods section for details), and then grow an isolation oxide layer at 300 °C using plasma-enhanced chemical vapor deposition (PECVD). The cross-sectional transmission electron microscopy (TEM) images confirm that the layer-1 (L1) and layer-2 (L2) VRRAM switching regions are formed by the titanium nitride (TiN)/hafnium oxide (HfO_x_)/platinum (Pt) structure, with TiN being the shared pillar electrode, and Pt sidewall being the other electrode (Fig. 1b). Then the 2D MoS_2_ transistors with local back gates are patterned on the middle plane, which demonstrates that additional transistor layers can be fabricated above the memory layer. Although the 2D MoS_2_ transistors are mainly leveraged for driving the VRRAMs in our design, this process provides the prospect for additional logic functionality and computational capability without consuming additional chip area. The centimeter-scale monolayer MoS_2_ is grown by CVD^18^, and transferred using a water-assisted transfer technique at 150 °C^52^, which reliably results in a MoS_2_ film on the target substrate already with the bottom-plane VRRAMs, back-gate electrodes, and gate dielectric. The MoS_2_ is then annealed in vacuum at 280 °C for one hour to enhance the quality and remove the adsorbates, followed by patterning of the source and drain electrodes, as shown by the cross-sectional TEM images (Fig. 1c). After another isolation oxide layer deposition, we repeat the processes for fabricating the two-layer 3D VRRAMs, and obtain the top-plane layer-3 (L3) and layer-4 (L4) VRRAMs, as shown by the cross-sectional TEM images (Fig. 1d). This demonstrates that the high-density memory layer can be further patterned on top of the transistor and logic layers. As such, we obtain the monolithic 3D integrated structure with a 2D MoS_2_ transistor driving four VRRAMs, i.e., a 1T–4R structure, with interlayer vias connecting different layers as shown in Fig. 1e, f. Both broader-view and zoom-in cross-sectional TEM images of the 1T–4R structure with elemental mapping are demonstrated in Supplementary Fig. 1, showing clear contrast among different device layers, conformal growth of materials, as well as smooth and intimate interfaces of the VRRAMs. The important fabrication processes with the optical image of the 2D MoS_2_ after transfer are shown in Supplementary Figs. 2 and 3 and explained in Supplementary Note 1. Such structure demonstrates the feasibility and versatility of fabricating 3D VRRAM planes on top of 2D FET planes, as well as fabricating 2D FET planes on top of 3D VRRAM planes.

Such integration of the bottom two-layer VRRAMs, middle MoS_2_ transistors, and top two-layer VRRAMs is feasible owing to the very low fabrication temperature and low thermal budget, with the highest temperature among all the processes being the PECVD isolation oxide growth at 300 °C. The low fabrication temperature also avoids the thermal stress issue for the interlayer vias. At such temperature, it will be very difficult to achieve high-quality CMOS logic, main memory, or flash memory devices. It is worth noting that while the growth temperature of 2D MoS_2_ is at 850 °C, this process is separated from other fabrication steps with a low-temperature transfer process, and will not affect the device performance. The *I*_D_–*V*_GS_ characteristics for 40 MoS_2_ FETs on the same chip demonstrate relatively small device-to-device variation (Supplementary Fig. 4a), and the *I*_D_–*V*_DS_ characteristics in Supplementary Fig. 4b show large enough drive currents and the capability to sustain large enough voltages that support VRRAM programming. This process also holds the potential for vertically stacking more memory or transistor layers towards a 1T–*n*R structure (Fig. 1f), for even higher integration density.

### 3D VRRAM characterization

**Fig. 2:**
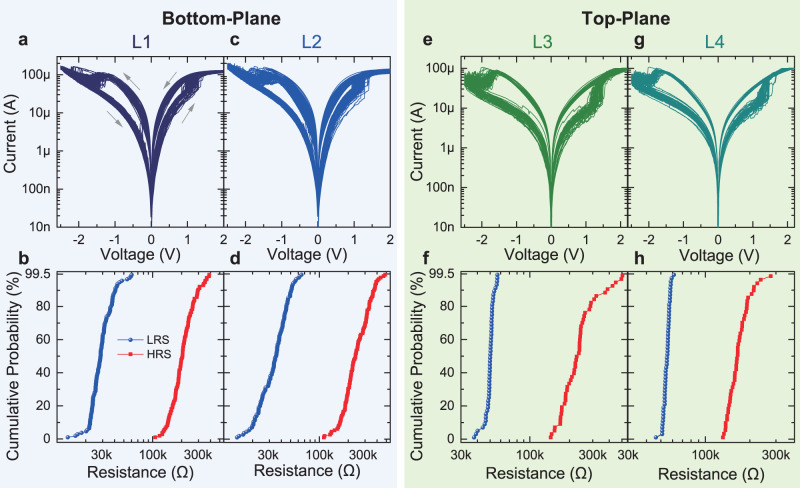
Electrical characterizations for each layer of VRRAM, with *V*_GS_ = 4 V and the compliance current provided by the MoS_2_ transistor. **a–d** The DC *I***–***V* sweeps (top) and the corresponding resistance distributions (bottom) of the bottom-plane two-layer VRRAMs, for **a–b** L1, and **c–d** L2 VRRAMs. The results show that the MoS_2_ transistors can reliably drive the VRRAM switching for multiple cycles. **e–h** The measurements of the top-plane two-layer VRRAMs shown in the same sequence as in **a–d**, for **e–f** L3, and **g–h** L4 VRRAMs.

We then characterize the electrical properties for each of the four layers of the 3D VRRAMs. During the measurement of the 1T–4R device, the 2D MoS_2_ transistor is turned on by applying proper gate voltages (*V*_GS_). The write/read voltages are applied to the drain electrode of the 2D MoS_2_ transistor, with the source electrode connected to the pillar (top) electrode of the VRRAM, the bottom electrode of the layer of VRRAM under test grounded, and the bottom electrodes of other layers floating. This forms a 1T–1R structure when measuring each layer of the VRRAM. The initial forming cycle for each layer of 3D VRRAM is performed below 3.5 V (Supplementary Fig. 5), and the following DC *I***–***V* sweeps show 50 cycles of switching for each layer (Fig. 2). We show that the 2D MoS_2_ transistors can successfully drive the VRRAMs, and limit the current during the set process, for both the bottom-plane 2-layer VRRAMs (Fig. 2a–d), and top-plane 2-layer VRRAMs (Fig. 2e–h). The resistance distributions for the high-resistance state (HRS) and low-resistance state (LRS) are similar among the four layers of VRRAMs, showing high uniformity. To demonstrate the effect of 2D MoS_2_ transistors on programming the VRRAMs, we also compare with the case when the set current is limited by the equipment, by increasing the transistor gate voltage, so that the transistor channel resistance is not the limiting factor of the compliance current (Supplementary Fig. 6). Although the switching window does not change significantly, the resistance values of the HRS and LRS both increase when using the MoS_2_ transistors as the current compliance, because the MoS_2_ FETs are more effective in limiting the current during the set operation^47^. The higher resistance states are advantageous for reducing the power consumption during the memory write/read operations.

### Uniformity of VRRAMs and 2D MoS_2_ FETs

**Fig. 3:**
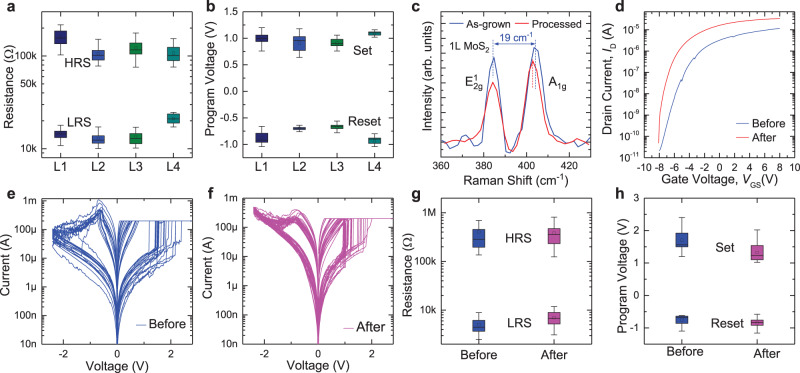
Characterization of the device uniformity. Measured statistics of **a** the HRS and LRS resistances, and **b** the set and reset voltages, for L1 to L4 VRRAMs from the 50 DC *I*–*V* sweeps in Fig. 2. **c** Raman spectra of the as-grown and processed monolayer MoS_2_, showing Raman mode softening for the A_1g_ peak. **d** *I*_D_–*V*_GS_ curves of a 2D MoS_2_ FET before and after the top-plane device fabrication. Comparison of the resistive switching characteristics for the same bottom-plane VRRAM device **e** before and **f** after the top-plane device fabrication. Statistics of **g** the LRS and HRS resistances, and **h** the set and reset voltages before and after the top-plane device fabrication, for the measurement results in **e–f**. In **a**, **b**, **g**, and **h**, the colored ranges in the box chart (25%–75%) show the interquartile range (IQR), which is between the first and third quartile of the data distribution, and the error bars show the range within 1.5 IQR.

For vertically stacked 3D structures, the uniformity of device properties among different layers is a critical metric to be investigated. The uniformity can be evaluated from the following aspects. First, the VRRAM device properties among the four layers should be uniform. We show the uniformity of the resistance distributions and programming voltages for L1 to L4 VRRAMs, using the measured DC *I*–*V* sweeps for multiple cycles (Fig. 3a, b). For each of the four layers of VRRAMs, over 10^6^ pulsed switching cycles can be obtained while maintaining a clear resistance separation (Supplementary Fig. 7). To verify the scalability of the technique, we have fabricated and measured 40 VRRAMs for all four memory layers (Supplementary Figs. 8–11). We extract the device-to-device variation in each VRRAM layer (Supplementary Fig. 12), and show the distribution of the worst-case On/Off resistance ratios and set/reset voltages from multiple cycles of measurements for each of the 40 VRRAMs (Supplementary Fig. 13).

Second, it is desirable that the fabrication of the top-plane devices does not adversely affect the bottom-plane devices. For validation, we measure the Raman spectra of the as-grown 2D MoS_2_ and the MoS_2_ after the transistor is fabricated and covered with 1.5 nm AlO_x_ and 10 nm HfO_x_, which shows uniform peak positions for E_2g_^1^ mode, and a softening effect in A_1g_ Raman mode (Fig. 3c). This suggests n-doping effect from the AlO_x_ layer^53^, because n doping will result in softening of the A_1g_ phonon mode, while E_2g_^1^ peak position is relatively insensitive to the doping effect. After the deposition of 100 nm SiO_2_ isolation layer, the Raman spectra of MoS_2_ can also be measured, which still shows a softening in A_1g_ mode (Supplementary Fig. 14). The peak separation between the E_2g_^1^ and A_1g_ Raman modes is ~19 cm^−1^ for as-grown MoS_2_ (Fig. 3c), which confirms that the CVD-grown MoS_2_ is monolayer^54^. We have also performed Raman mapping for the MoS_2_ film before and after the oxide deposition and other processing, and the results show relatively uniform 2D materials (Supplementary Fig. 15). The *I*_D_-*V*_GS_ curves of a 2D MoS_2_ FET also demonstrate that the fabrication of top-plane VRRAMs does not adversely affect the 2D MoS_2_ underneath (Fig. 3d). A negative shift in threshold voltage, and an increase in *I*_D_ and thus a higher driving capability is observed, which can also be attributed to the n-doping effect of the AlO_x_ isolation layer, and is consistent with the Raman measurements and the previous report^55^.

Furthermore, we measure the identical VRRAM on the bottom plane before and after the fabrication of the middle-plane MoS_2_ FETs and the top-plane VRRAMs, using DC *I*–*V* sweeps (Fig. 3e, f). The uniformity of distributions for resistances and program voltages (Fig. 3g, h) suggest the feasibility for vertical stacking of more transistor or VRRAM planes, without damaging the performance of the bottom-plane devices. Additional measurements for three other bottom-plane VRRAMs have been performed, further confirming that the VRRAMs remain functional and relatively uniform after the upper-plane device fabrication (Supplementary Fig. 16). Some differences between the resistive switching curves or resistance states before and after the upper-plane device fabrication can be attributed to the intrinsic cycle-to-cycle variations of VRRAMs. Such high uniformity in the monolithic 3D structure benefits from the low fabrication temperature (≤300 °C).

### Multi-level VRRAMs

**Fig. 4:**
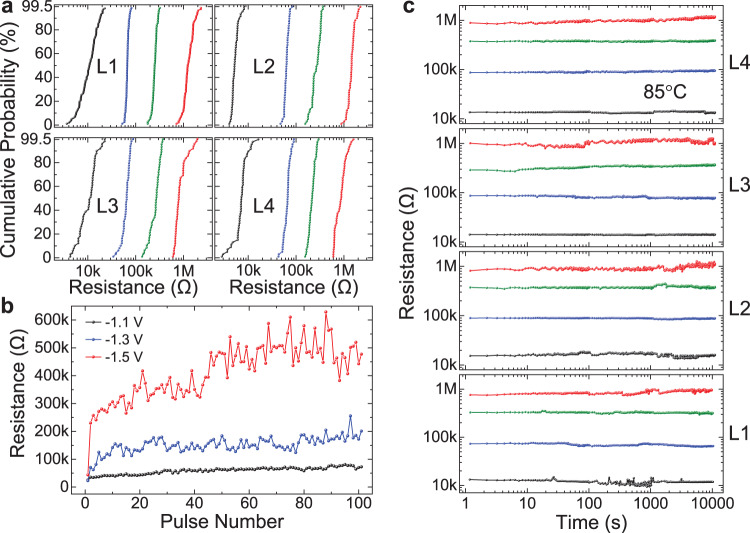
Multi-level VRRAM measurements. **a** Resistance distribution of four layers of VRRAMs, each showing four resistance states, realized by tuning the reset voltages. The black lines represent the LRS after the set operation, and the blue, green, and red curves show distributions for increasing resistance states, respectively, extracted from 50 cycles of *I*–*V* measurements. **b** Resistance modulation by increasing reset pulse numbers, using pulses with the rise time of 20 ns, fall time of 20 ns, and pulse width of 60 ns, at different voltage pulse amplitudes. **c** Retention measurements at 85 °C for the four layers of VRRAMs, with each layer of VRRAM showing four stable resistance states. The black, blue, green, and red data points and curves show the resistance states with increasing resistances.

We also verify the multi-level programming and sensing capability of the fabricated 3D VRRAMs, to demonstrate even higher memory density. The measurements are conducted using both DC voltage sweeps and voltage pulse measurements. By using different reset stop voltages during the DC voltage sweeps, we obtain four distinguishable resistance states for all four layers of VRRAMs, showing consistent resistance states (Fig. 4a). More resistance levels up to eight can be achieved in the same four layers of VRRAMs by carefully selecting the DC sweep voltages (Supplementary Fig. 17), which is useful for circumstances where some resistance distribution overlap among the different resistance states can be tolerated and the high memory density is essential. In addition, the resistance states can be continuously tuned by applying increasing numbers of reset voltage pulses, with larger voltage pulses leading to higher resistance states and having a larger resistance tuning range (Fig. 4b). We measure the retention property at 85 °C for all four layers of VRRAMs^27,46,48^, with each layer of VRRAM showing four stable resistance states with negligible degradation up to 10,000 s (Fig. 4c). Therefore, the 1T–4R structure stores 16 different resistance states in total. Furthermore, multiple resistance states can also be obtained through applying different *V*_GS_ on the 2D MoS_2_ FETs during the set process, with a larger *V*_GS_ leading to a smaller LRS resistance (Supplementary Fig. 18), because a larger *V*_GS_ can supply a larger current through the VRRAM during the set process, and can lead to a filament with a larger lateral size, which is consistent with previous RRAM models^56,57^. These two different techniques to tune the memory states provide more possibilities for the multi-state memory that increases the storage density. This can be achieved because the MoS_2_ FET works not only as an access switch, but also as a controller of the total current allowed during the set process of the VRRAMs. Such demonstration of the combination of multi-level cells and vertically stacked 3D VRRAMs provides unique opportunities for ultra-high density memory, as well as flexible and diverse device properties for computing systems.

### Cross-layer modeling of the monolithic 3D structure

**Fig. 5:**
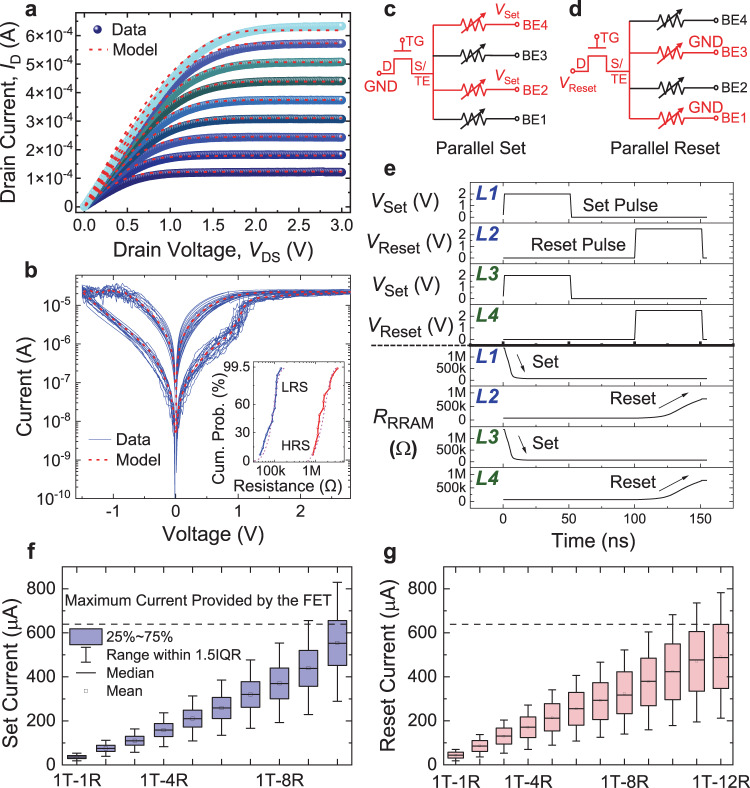
Parallel programming capability modeling for the 1T–*n*R structure. **a** Measured *I*_D_*–V*_DS_ characteristics of a 2D MoS_2_ FET at different *V*_GS_, with fitting to the PTM model shown by the red dashed lines. **b** Measured representative resistive switching characteristics of a VRRAM device, with fitting to the compact model shown by the red dashed line. *Inset*: The resistance distribution and fitting for the HRS and LRS states of the VRRAM. Schematic illustrations of the circuit for the parallel **c** set and **d** reset processes. **e** HSPICE transient simulation for programing the 1T–4R structure, where two parallel set operations are performed on the L1 and L3 VRRAMs, and then two parallel reset operations are performed on the L2 and L4 VRRAMs. Simulation of the required switching currents for the parallel **f** set and **g** reset operations, with VRRAM resistance variations taken into consideration. The colored ranges in the box chart (25%–75%) show the interquartile range (IQR), and the error bars show the range within 1.5 IQR. The MoS_2_ FET can drive up to 8 layers of VRRAMs during the set process and up to 9 layers during the reset process. The variation in set voltage results in a more strict limit during the parallel set process.

To further evaluate the system-level performance gain, we develop the device model using HSPICE for our 1T–*n*R memory cells based on the measurement data. The measured *I*_D_*–V*_DS_ characteristics of the MoS_2_ FETs are fitted using a 130 nm BSIM4 Predictive Technology Model (PTM) (Fig. 5a)^58^, with the fitting parameters listed in Supplementary Table 1a. By fitting to the *I*_D_*–V*_DS_ behavior, we obtain the device model that can be utilized for the following circuit-level simulation. Meanwhile, the measured set and reset characteristics, and the resistance distributions in HRS and LRS of the VRRAM cells are fitted using the RRAM compact model (Fig. 5b)^59^, with the fitting parameters summarized in Supplementary Table 1b and explained in Supplementary Note 2.

Based on the MoS_2_ FET model and the VRRAM model, we further simulate the parallel programming of the VRRAMs on the same pillar in the 1T–*n*R scheme. We limit the set compliance current to be around 20 μA by controlling the gate voltage of the MoS_2_ transistor, which can result in a higher LRS state resistance^56,57^. The set/reset power is down to 20 μW and 30 μW, respectively, which is beneficial for increasing the energy efficiency and parallel programming capability. By properly biasing the FET and the VRRAMs, we can perform either parallel set (Fig. 5c) or parallel reset operations (Fig. 5d) in the vertical direction. HSPICE transient simulation in Fig. 5e demonstrates a representative scenario where the L1 and L3 VRRAMs are first set to LRS in parallel, and then the L2 and L4 VRRAMs are reset to HRS in parallel. The VRRAM resistance states also affect the parallel driving capability. With a fixed HRS resistance, a larger LRS resistance may lead to the overlapped LRS and HRS when non-ideal effects are present, thus resulting in the bit error. Meanwhile, a severely low resistance value of LRS will increase the required reset current. As the number of parallelly programmed VRRAMs increases, we raise the gate voltages on the MoS_2_ FET to enlarge the drive currents. The simulated parallel switching dynamics for different 1T–*n*R schemes in Supplementary Fig. 19 show that the parallel programing capability is successfully improved by controlling the gate voltage.

We then perform measurements on the parallel set and reset behaviors for two of the VRRAM layers in the 1T–4R structure (Supplementary Fig. 20), using the schemes as shown in Fig. 5c, d. The intrinsic device-to-device variation could complicate the parallel programming operation of VRRAMs due to voltage and current division effects. The detailed switching dynamics are modeled in Supplementary Figs. 21 and 22, and discussed in Supplementary Note 3. If one VRRAM shows the set transition earlier than the others, the transistor not only should provide enough current for the parallel set operation, but also needs to have a relatively small On-state resistance compared with the parallel resistance of VRRAMs in LRS states, to ensure enough voltage drop on the VRRAMs to enable the programming of the other VRRAM. Nevertheless, by increasing the applied voltage after the set transition of the first VRRAM, parallel set behavior with a 2D MoS_2_ transistor driving two layers of VRRAMs has been experimentally demonstrated, with the second VRRAM programmed at a larger voltage compared with the first VRRAM (Supplementary Fig. 20). It is also observed that the parallel reset behavior is barely affected by the device variation, because the reset of one VRRAM can make the reset of another VRRAM easier due to a larger voltage drop across the VRRAMs.

The parallel programming capability in the vertical direction is determined both by the amount of drive current provided by the MoS_2_ FETs and the intrinsic device-to-device variation of VRRAMs. We first model the drive current required for simultaneously switching different numbers of VRRAM layers during the set (Fig. 5f) and reset (Fig. 5g) processes, and compare with the current that can be provided by the MoS_2_ FETs. With the VRRAM variations taken into consideration, we show that for the set process, the MoS_2_ FET can drive up to 8 layers of VRRAMs; and for the reset process, it can drive up to 9 layers of VRRAMs in parallel in the 1T–*n*R configuration. The parallel driving capability can be further improved by decreasing the contact resistance, using 2D semiconductors with a higher carrier mobility, or constructing gate-all-around transistors by stacking 2D nanosheets^60^.

**Fig. 6:**
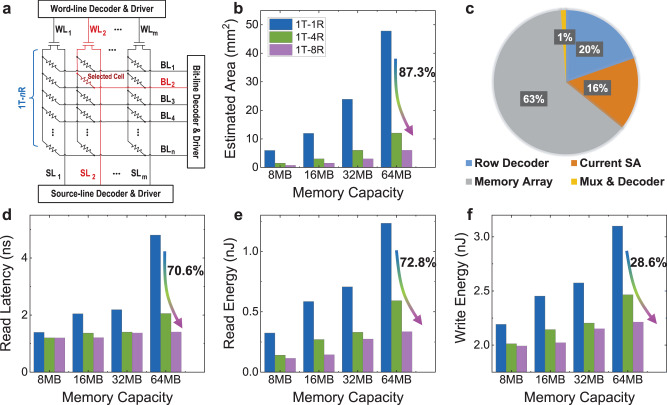
Simulation of the memory system performance based on the monolithic 3D integrated MoS_2_ FETs and VRRAMs. **a** Illustration of the circuit simulation framework for the 1T–*n*R structure in NVSim. **b** Comparison of the estimated area of the memory systems using the 1T–1R, 1T–4R, and 1T–8R structures, for different capacity configurations (8–64 MB). **c** The area breakdown for the 1024 × 1024 1T–4R VRRAM array and the peripheral circuits. Comparison of the **d** read latency, **e** read energy, and **f** write energy among the 1T–1R, 1T–4R, and 1T–8R structures, during the parallel read or write with 1024-bit I/O bandwidth, for the memory capacity of 8–64 MB.

To reveal the benefits of using such 1T–*n*R monolithic 3D structure in memory systems, we perform circuit-level modeling at 65 nm node using NVSim^61^, a circuit-level performance simulation tool for nonvolatile memories. The 1T–*n*R circuit and architecture to be evaluated are illustrated in Fig. 6a^38,40,42^, with the detailed configuration summarized in Supplementary Table 2. According to the measured device parameters, we perform architectural modifications in NVSim to obtain the area, latency, and energy of the memory array with different memory capacity (8–64 MB), and compare the performance among 1T–1R and 1T–*n*R configurations.

A straightforward advantage for using 1T–*n*R instead of 1T–1R configuration is the higher memory density for the same footprint. For a memory array with 1024 × 1024 memory cells and necessary peripheral circuits, the memory array area is compared among the 1T–1R and 1T–*n*R configurations, showing that the 1T–4R and 1T–8R configurations result in up to 74.8% and 87.3% reduction in area, respectively, when compared with the 1T–1R configurations for the same memory capacity (Fig. 6b). This is due to the efficient use of the vertical direction for stacking. Here the extra peripheral circuits including the bit-line multiplexers (Mux), column decoders, and current sense amplifiers (SA) for the 1T–*n*R design have been considered in the simulation, as detailed in Supplementary Note 4 and Supplementary Fig. 27. The area breakdown specified in Fig. 6c reveals that the memory array accounts for the majority of the area for the whole system, which is up to 63% for a 1024 × 1024 VRRAM array based on the 1T–4R cells. Compared with the conventional 1T–1R design, the extra peripherals account for only ~0.8% and ~1.6% of the area overhead for the 1T–4R and 1T–8R arrays, respectively. The significantly reduced area by using the 1T–*n*R 3D stacking technique can effectively enhance the on-chip memory capacity and reduce the manufacturing cost.

Besides the reduced area, additional benefits in the read latency and read/write energy of the 1T–*n*R configuration have also been analyzed (Fig. 6d–f). Such benefits rely on the largely reduced length in signal/data transfer through the vertical interlayer vias instead of the long planar paths, as well as the reduced path from the input/output (I/O) ports to the memory array, because of the reduced chip size compared with the 1T–1R array with the same capacity. We perform the evaluations by reading and writing the identical data in parallel, with the fixed 1024 I/O ports, for the 1T–1R and 1T–*n*R configurations. Compared with the 1T–1R configuration, significant improvement is achieved in the read latency, with up to 57.2% and 70.6% reduction for the 1T–4R and 1T–8R configurations, respectively (Fig. 6d). Consistent with the reduced read latency, the read energy is reduced by up to 72.8% (Fig. 6e), and the write energy consumption is also reduced by up to 28.6% (Fig. 6f), for the 1T–8R configuration. The write latency is barely improved, since it is dominated by the 50 ns set/reset write pulse signal in the simulation. With more VRRAM layers stacked and an even higher memory capacity, the area, latency, and energy consumption can be further reduced for a fixed memory size (Supplementary Table 3).

## Discussion

Specifications of our work as well as other representative monolithic 3D integration structures are summarized in Supplementary Table 4. Our work shows the fabrication temperature below 300 °C, which benefits from the low-temperature transfer of the 2D materials and the low-temperature fabrication of the VRRAMs. The low fabrication temperature ensures a low thermal budget, which allows the bottom-plane device performance to be unaffected by the top-plane device fabrication, and thus allows further vertical stacking of additional logic and memory layers. Furthermore, our structure uses the 3D VRRAM to achieve a higher storage density than the planar memory array.

We also summarize the characteristics of different memory technologies and different VRRAM devices, in Supplementary Tables 5 and 6, respectively. We demonstrate that the VRRAMs in this work are suitable for the monolithic 3D integration structure, with up to 8 memory states per layer of VRRAM for further increasing the memory density, low programming latency down to 60 ns, small cell size of 33 nm × 5 nm (sidewall), and scalability up to 512 Mb VRRAM array. Furthermore, up to 8 layers of VRRAMs with self-selecting property have been experimentally demonstrated in previous work^37,62–65^, which are well compatible with such monolithic 3D integration structure, and hold high potential to further enhance the memory density and reduce the power consumption for these monolithic 3D systems. These results demonstrate that the monolithic 3D integrated structure with the 1T–*n*R configuration is a promising methodology towards high-density and large-capacity on-chip memory and in-memory computing systems with fast memory access and low power consumption. Such monolithic 3D system can be complementary to CMOS circuits, and the low fabrication temperature may allow their integration on top of CMOS circuits for higher memory density and additional functionality.

Recent progresses on wafer-scale growth and transfer of 2D materials with high uniformity show the potential for large-scale monolithic integration of 2D FETs^66,67^. We have scaled down the 2D MoS_2_ transistors, and experimentally demonstrate transistors with channel length down to 100 nm, and subthreshold swing down to 100 mV/dec (Supplementary Figs. 23 and 24). In our experiment, we use noble metal Pt as the bottom electrode of the VRRAMs. Towards better CMOS compatibility, we have further fabricated 2-layer VRRAMs using a TiN/HfO_x_/Ti structure, which also demonstrates resistive switching properties (Supplementary Fig. 25), consistent with previous reports^37,40,46^. While the vertically stacked structure could lead to an increase in interlayer capacitance, simulation shows that the increase in latency is minimal (Supplementary Fig. 26). Power dissipation in monolithic 3D systems could be more complicated than planar structures, and could potentially lead to issues in 3D integration. Therefore, we further simulate the thermal dissipation and temperature in the 1T–*n*R array using the 3D-ICE emulator^68^, and show that the temperature increase is tolerable for reasonably large parallel data transfer, as detailed in Supplementary Figs. 28 and 29. Furthermore, from our simulation, when the amount of transistor is fixed in a memory array, the memory size will increase due to vertical stacking of more memory layers, while the area, read/write delay, and read/write energy show minimal change (Supplementary Table 7).

In summary, aimed at the memory density problem in monolithic 3D integration systems, we demonstrate the monolithic 3D integration of 2D MoS_2_ FETs and 3D VRRAMs, into a 1T–4R structure with five device layers, which can enable high-density and low-power on-chip memories. Such integrated structure is fabricated below 300 °C, which allows the vertical stacking of the top-plane devices without affecting the bottom-plane device performance. Measurements show the uniformity of VRRAM performance among the different device layers, as well as the consistency of the MoS_2_ FET and VRRAM characteristics before and after the top-plane fabrication. The low fabrication temperature allows the continuous vertical stacking of transistor planes and memory planes at any order. Each VRRAM cell can be programmed into four stable resistance levels, further enhancing the memory density. Simulation results reveal that the 1T–*n*R structure can largely reduce the chip area, read latency, and read/write energy consumption compared with 1T–1R structures. The 2D MoS_2_ transistors with high On/Off ratio can effectively suppress the leakage current, and allow scaling towards large-scale memory arrays. Such monolithic 3D integration paves the way for a fine-grained integration of logic and high-density on-chip memory towards the high-bandwidth and low-power memory and computing systems.

## Methods

### Growth of 1 L MoS_2_

The MoS_2_ is grown using CVD in a furnace with 2-inch inner diameter. The continuous monolayer MoS_2_ film is synthesized from solid S and MoO_3_ precursors with the assistance of perylene-3,4,9,10 tetracarboxylic acid tetrapotassium salt (PTAS)^18^. The substrate with PTAS on 300 nm SiO_2_ on Si has a size of around 2 cm × 2 cm size, and is placed facing down on an alumina crucible containing ~ 1 mg of MoO_3_ and placed at the heating center of the furnace. Then ~200 mg of solid S is placed upstream in a quartz boat from MoO_3_. Followed by pumping the tube to vacuum, 500 sccm N_2_ flows through the tube to reach atmospheric pressure (760 Torr). The temperature during the growth is kept at 850 °C, resulting in continuous monolayer MoS_2_ on the chip.

### Material characterization

The Renishaw inVia confocal Raman microscope is applied to characterize the Raman spectra of the 1 L MoS_2_, with a continuous wave laser at 532 nm wavelength. TEM samples of transistors and each layer of VRRAM are prepared by a focused ion beam/scanning electron microscopy dual beam system (TESCAN, CZ). The TEM (FEI, USA) operates at 200 kV in bright-field TEM mode when used for imaging.

### Transfer of MoS_2_ on the devices

The MoS_2_ is transferred using a water-assisted transfer process^52^. About 4 g polystyrene (PS) is dissolved into 20 mL toluene, and spin coated onto the 1 L MoS_2_ on the growth substrate at 3000 rpm, and baked at 80 °C. About 1 mL deionized water is then injected at the edge to make the material detach from the substrate. The PS with the 2D material is then gently lifted up and the residual water is carefully blown dry, and then baked at 150 °C. After the PS is removed in solution, the 2D material is transferred to the target substrate.

### Device fabrication

Fabrication of the monolithic 3D integrated structure starts with the bottom-plane 3D VRRAMs. Following photolithography, Ti (3 nm)/Pt (30 nm), and ~100 nm SiO_2_ are sputter deposited. Such process is repeated twice to result in the two plane electrodes for the VRRAMs. No via etching process is needed after the deposition because the shape of the bottom electrode has been defined by lithography. Then thermal atomic layer deposition (ALD) is used to grow 5 nm HfO_x_ as the VRRAM switching layer, which ensures conformal coating on the sidewalls of the electrodes. The shared pillar electrode is then patterned using photolithography and sputter deposition. This forms the bottom-plane two-layer 3D VRRAMs. Isolation layer of 100 nm SiO_2_ is grown by plasma-enhanced CVD (PECVD) at 300 °C. For the middle-plane MoS_2_ FETs, the local back gates with 5 nm titanium (Ti) and 35 nm gold (Au) are first patterned, and 55 nm HfO_x_ is grown by ALD as the gate dielectric. The ILVs between the bottom-plane and middle-plane are then patterned using reactive ion etching. The CVD MoS_2_ is then transferred at low temperature (below 150 °C), followed by vacuum annealing at 280 °C for 1 h. Then the MoS_2_ is etched into the designed shape, and then photolithography (for 0.8 μm channel device) or electron beam lithography has been used to pattern the source/drain metal contacts, followed by evaporation of with 5 nm Ti and 35 nm Au. Here the contact to MoS_2_ is Ti, and the Au is used as a capping layer to avoid oxidation. Other contact materials such as nickel (Ni), scandium (Sc^69^), and bismuth (Bi^16^), etc., have also been demonstrated in literature, showing favorable contact properties. Then 1.5 nm aluminum (Al) is evaporated, and then exposed to air so that it quickly oxidizes into substoichiometric AlO_x_, which is in direct contact with MoS_2_ and forms the seed for the following processes. ALD HfO_2_ and PECVD SiO_2_ isolation layers are then grown, followed by top-plane two-layer 3D VRRAM fabrication using the same process as the bottom-plane VRRAMs. Etching of ILV and the oxide on the probing pads are performed when necessary. This forms the monolithically 3D integrated vertical structure of 2D MoS_2_ FETs and 3D VRRAMs, as confirmed by the cross-sectional transmission electron microscopy (TEM) images (Fig. 1).

### Electrical characterization

Electrical measurements are performed using a Keithley 4200A-SCS semiconductor characterization system connected to a probe station. All measurements are conducted in atmospheric pressure at room temperature except the retention measurement which is at 85 °C. Source measurement units (SMUs) are used in quasi-DC measurements, and the pulse measurement units (PMUs) are used for the pulsed measurements. During pulse measurements, a minimum pulse width of 60 ns is used.

### Supplementary information

Supplementary_Information

## Data Availability

All data needed to evaluate the conclusions in the paper are present in the paper and/or the Supplementary Information. Other relevant data of this study are available from the corresponding author upon reasonable request.

## Code availability

All relevant code or algorithm of this study are available from the corresponding author upon reasonable request.
