# Supplementary material for: Monolithic 3D integration of 2D transistors and vertical RRAMs in 1T–4R structure for high-density memory
Source: Nat Commun. 2023 Sep 23;14:5952. doi: 10.1038/s41467-023-41736-2 (PMC10517937; doi:10.1038/s41467-023-41736-2)
Supplement: Supplementary file 1 — Supplementary_Information [file 41467_2023_41736_MOESM1_ESM.pdf]

Supplementary Information

Monolithic 3D Integration of 2D Transistors and Vertical RRAMs in 1T–4R Structure for High-Density Memory

Maosong Xie[1,†], Yueyang Jia[1,†], Chen Nie[2], Zuheng Liu[1], Alvin Tang[3], Shiquan Fan[4], Xiaoyao Liang[2], Li Jiang[2,5,6], Zhezhi He[2,*], Rui Yang[1,2,7,*]

[1]*University of Michigan – Shanghai Jiao Tong University Joint Institute, Shanghai Jiao Tong University, Shanghai, China*
[2]*School of Electronic Information and Electrical Engineering, Shanghai Jiao Tong University, Shanghai, China*
[3]*Department of Electrical Engineering, Stanford University, CA, USA*
[4]*School of Microelectronics, Xi'an Jiaotong University, Xi'an, Shaanxi, China*
[5]*MoE Key Lab of Artificial Intelligence, Shanghai Jiao Tong University, Shanghai, China*
[6]*Shanghai Qi Zhi Institute, Shanghai, China*
[7]*State Key Laboratory of Radio Frequency Heterogeneous Integration, Shanghai Jiao Tong University, Shanghai, China*

[†]Equal Contribution.
[*]Corresponding authors. Email: zhezhi.he@sjtu.edu.cn, rui.yang@sjtu.edu.cn

Supplementary Notes

1. Fabrication process

Fabrication of the monolithic three-dimensional (3D) structure starts from the fabrication of the bottom-plane 2-layer 3D vertical resistive random-access memories (VRRAMs) and the isolation layers (Supplementary Fig. 2a–2b), and then the middle-plane bottom-gated two-dimensional (2D) molybdenum disulfide (MoS_2) field-effect transistors (FETs) (Supplementary Fig. 2c), and finally the top-plane 2-layer 3D VRRAMs. The detailed fabrication processes and parameters are described in the Methods section of the main text. The final monolithic 3D integrated structure is shown in Supplementary Fig. 2d, showing the 4 layers of VRRAMs (L1–L4), formed between the edges of 4 bottom electrodes and the same shared pillar electrode. The access transistor is in the middle plane, which is different from complementary–metal–oxide–semiconductor (CMOS) transistors that usually need to be fabricated in the front end of the line, and are located at the bottom plane.

2. RRAM model and fitting parameters

The RRAM is modeled by considering the evolution of gap size as a function of time and external electric field, and the resistance variation is fitted by a Gaussian random parameter δ_{g0}. Under the voltage stress, the change rate of gap size will follow[2]:

$$\frac{dg}{dt} = -v_0 \times \exp\left(\frac{-E_a}{kT}\right) \times \sinh\left(\gamma \mathbf{E} \frac{a_0 q}{kT}\right) \tag{1}$$

where dg/dt is the change rate of gap size, v_0 is related to the lattice vibration rate, E_a is the energy barrier for the generation and recombination of vacancies, k is the Boltzmann constant, T is the temperature, $\mathbf{E}$ is the electric field, a_0 is the lattice constant, q is the unit electron charge. γ is the field enhancement factor given by $\gamma = \gamma_0 - \beta \cdot (g/g_1)^{\alpha}$, where β, g_1, and α are the fitting parameters. The change of gap size during a time interval is then:

$$dg = \int \left(\frac{dg}{dt} + \frac{\delta_{g0}}{1 + \exp\left[\frac{T_{crit} - T}{T_{smooth}}\right]} \times \chi(t) \right) dt, \tag{2}$$

where T_{crit} and T_{smooth} are the fitting parameters reflecting the temperature sensitivity of variations, and $\chi(t)$ is a randomly generated zero-mean Gaussian sequence.

The current through the RRAM is calculated as a function of gap size using:

$$I = I_0 \times \exp\left(-\frac{g}{g_0}\right) \times \sinh\left(\frac{V}{V_0}\right), \tag{3}$$

where I_0, g_0, and V_0 are fitting parameters extracted from the experimental data, which are related to the amount of current, resistance window, and nonlinearity, respectively. The fitted parameters for our measured RRAM data are shown in Supplementary Table 1b.

3. Parallel programming with VRRAM variation effect

When VRRAM device variation is considered, the parallel programming is more challenging, especially during the set process. As shown in Supplementary Fig. 21, when we perform parallel set operation on two VRRAMs in the 1T–4R structure, if the VRRAMs have different set voltages due to variation, then the VRRAM with a smaller set voltage will be turned into LRS first. This results in a decrease of the parallel VRRAM resistance, leading to a smaller voltage drop on the VRRAMs due to the voltage division with the On-state transistor. Therefore, to enable the set transition, the other VRRAM either requires longer time (Supplementary Fig. 21d–21f), or a larger total applied voltage (Supplementary Fig. 21g–21i). Therefore, to properly set the other VRRAM, it is desirable to have a relatively high LRS state for the VRRAM and a low On-state resistance for the transistor. This can ensure that the voltage drop across the parallel VRRAMs is higher than the set voltage of the second VRRAM.

We compare the simulation results for the parallel set and reset behaviors, and find that the parallel reset can usually be reliably achieved although there is device variation, as shown in Supplementary Fig. 22f–22j, because when the parallel VRRAM resistance increases after the reset transition of one VRRAM, the voltage across the parallel VRRAMs increases due to voltage division with the transistor. The current in another VRRAM then increases, and the reset of another VRRAM is easier. When performing parallel set operation, because the parallel VRRAM resistance decreases after the set transition of one VRRAM, the voltage across the parallel VRRAMs decreases instead, due to voltage

division with the transistor, which then reduces the current in another VRRAM, and makes the set of the other VRRAM more difficult (Supplementary Fig. 22a–22e). In the experiment, by increasing the applied voltage after the set transition of the first VRRAM, we demonstrate measurements of both parallel set and reset of two VRRAMs in a 1T–4R structure (Supplementary Fig. 20).

4. Peripheral circuits for 1T–*n*R array simulation

To evaluate our fabricated device from the architecture perspective, we inherit the memory organization in Ref. [24] with mandatory modifications, and then utilize its circuit-level modeling tool for performance evaluation. Our modifications to support the 3D stacking with our 1T–*n*R memory includes bit-line multiplexers and column decoders. Further descriptions are specified in the following to justify the necessity of added components and their functionalities.

In Supplementary Fig. 27a, we show the internal organization (hierarchy) of a VRRAM used in Ref. [24], which includes the bank, mat and sub-array. The sub-array is at the lowest hierarchy (elementary block) of memory. We integrate the 1T–*n*R structure into the conventional 1T–1R memory architecture, with minimum modification (*i.e.*, modifying the sub-array only). For the circuit-level schematic of the sub-array with 1T–4R cells depicted in Supplementary Fig. 27b, there are horizontal Word-Lines (WLs) and vertical Bit-Lines (BLs) and Source-Lines (SLs) in the sub-array. Instead of using the single pair of BL and SL for the 1T–1R, we insert another three BLs for 1T–4R cells as they are five-terminal structures. Note that the inserted BLs are shared by the 1T–4R cells within the same column.

We do not attempt to modify the memory accessing mechanism out of the sub-array. Therefore, while the bandwidth of Input/Output (I/O) interface is not changed compared with its 1T–1R counterpart, additional bit-line multiplexers have to be inserted to precisely select the specific BLs corresponding to the different cells within the same 1T–4R.

Corresponding to that, additional decoders are required as well to convert the column address to the code for controlling the BL multiplexer, to fulfill the BL selecting task.

Supplementary Figures

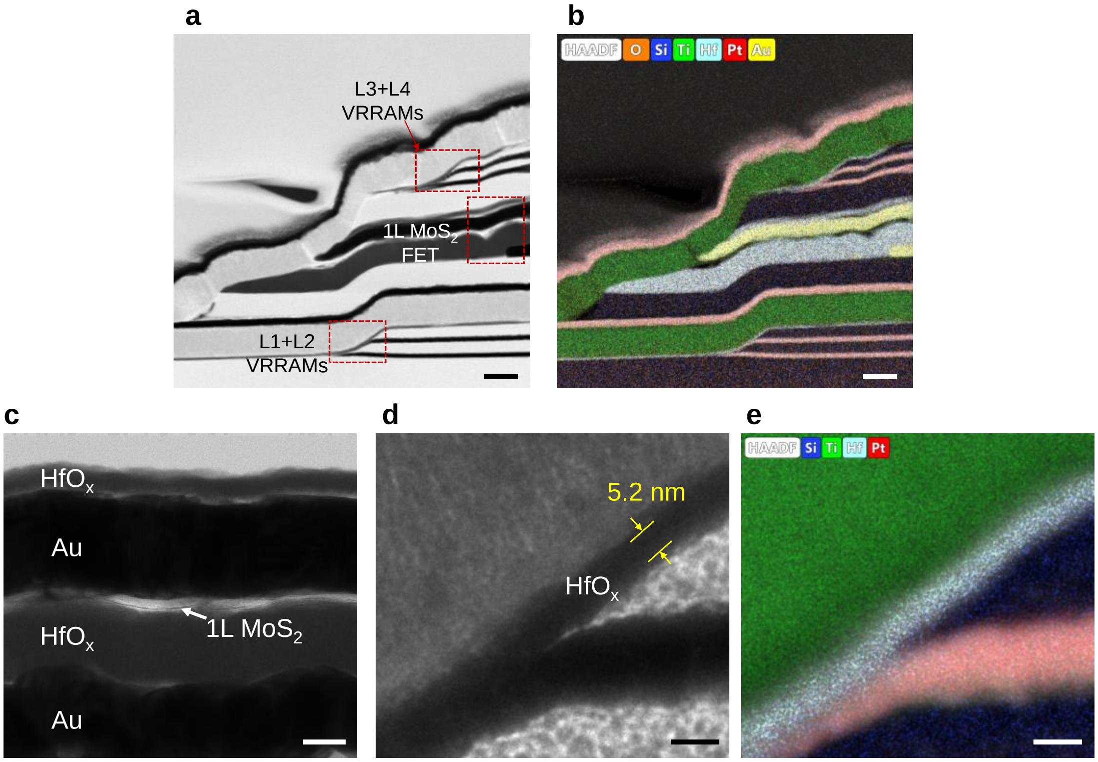

Figure S1. Overview and zoom-in cross-sectional transmission electron microscopy (TEM) images of the 1T–4R structure with the corresponding elemental mapping. a–b The cross-sectional **a** TEM image and **b** corresponding elemental mapping of the whole 1T–4R structure, with each plane of device highlighted with a red dashed box, showing the monolithic 3D integration of VRRAMs and 2D transistors. *Scale bars*: 100 nm. **c** The zoom-in TEM image of the MoS_2 transistor region. *Scale bar*: 20 nm. **d–e** The zoom-in **d** TEM image and **e** corresponding elemental mapping of a VRRAM switching region. The measured thickness of HfO_x is ~5.2 nm. *Scale bars*: 10 nm.

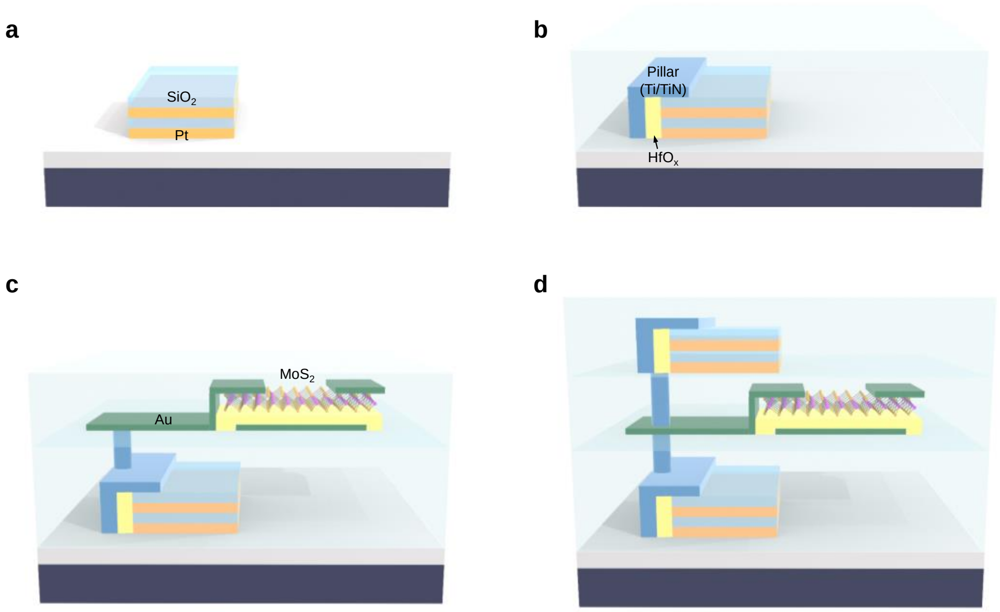

Figure S2. Schematic illustration of some key fabrication processes. a Two layers of VRRAM bottom electrodes and the isolation layers between the electrodes. **b** The HfO_x switching layer and pillar (top) electrode of 3D VRRAMs. **c** Isolation layer, interlayer via, FET back gate, HfO_2 gate dielectric, MoS_2 transfer, and S/D contact electrodes. **d** Isolation layer, interlayer via, and top 2-layer 3D VRRAMs.

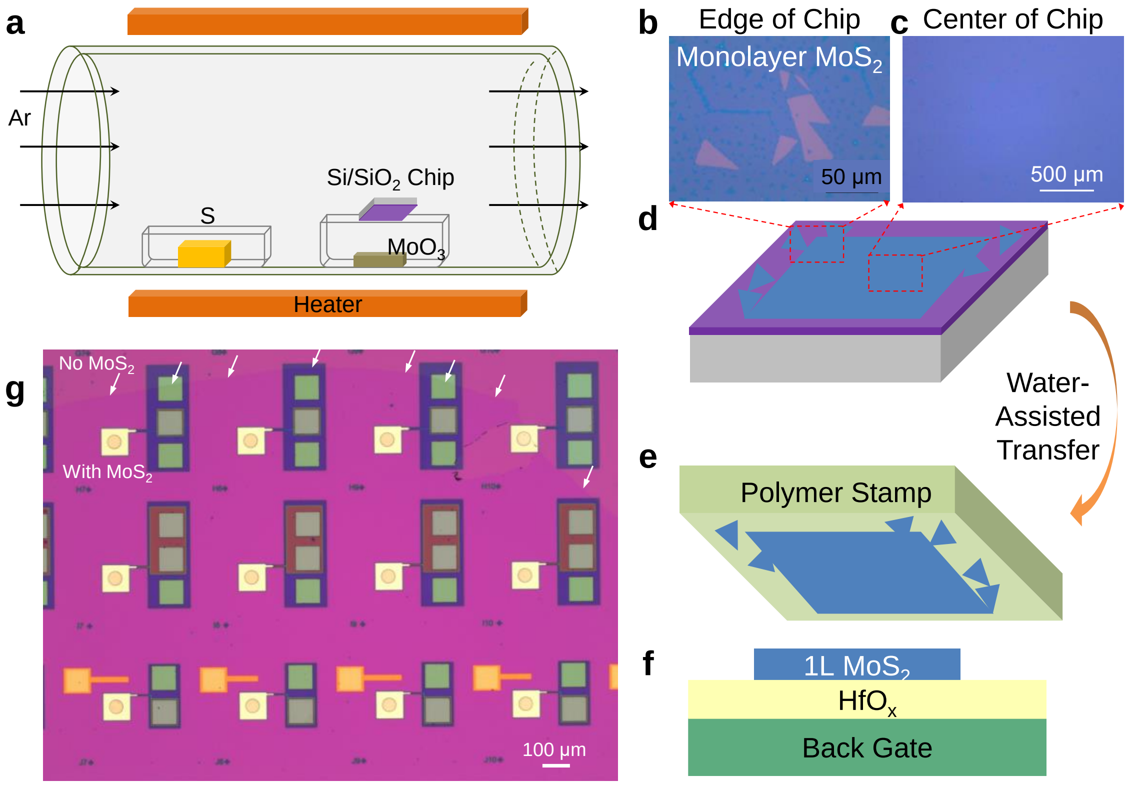

Figure S3. Growth and transfer of monolayer MoS_2. a Schematic illustration of the chemical vapor deposition (CVD) growth with the furnace, solid S and MoO_3 precursors, and Ar environment. **b–d** CVD-grown monolayer MoS_2, including **d** schematic illustration of the Si/SiO_2 substrate after MoS_2 growth, **b** optical image of the triangular single crystals close to the edge of the chip as shown by the dashed box in **d**, and **c** optical image of the continuous MoS_2 film close to the center of the chip as shown by the dashed box in **d**. The area of the continuous film region on the chip is centi-meter scale, which is used for the fabrication of MoS_2 transistors. **e** Illustration of the water-assisted transfer process onto the polymer stamp, and then onto the pre-patterned substrate with devices. **f** Cross-sectional illustration of the MoS_2 transferred onto the substrate with local back gates and HfO_x gate dielectric, for forming the MoS_2 transistors in the middle plane. **g** Optical image of the chip after the MoS_2 transfer, with the white arrows showing the edge of the transferred film, so that the top region has no MoS_2, and the middle and bottom region has the 2D MoS_2 film. The 3D VRRAMs have already been fabricated in the bottom plane.

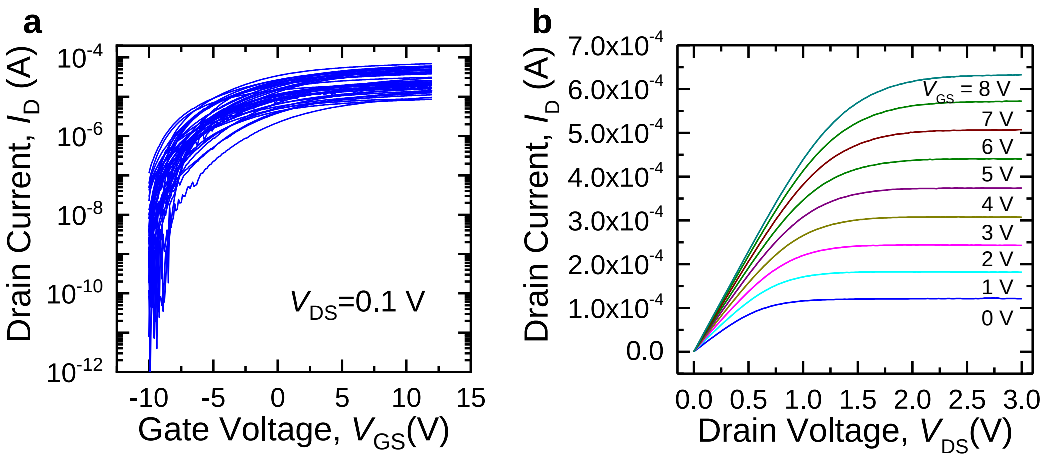

Figure S4. Electrical characterization of the 2D MoS_2 FETs. a I_D-V_{GS} curves of 40 different 2D MoS_2 FETs with local back gates, showing relatively small device-to-device variations. **b** I_D-V_{DS} characteristics measured at different V_{GS}, for a MoS_2 FET with W/L=100 μm/0.8 μm, which can reach up to 0.64 mA at V_{GS}=8 V, ensuring enough current for VRRAM switching.

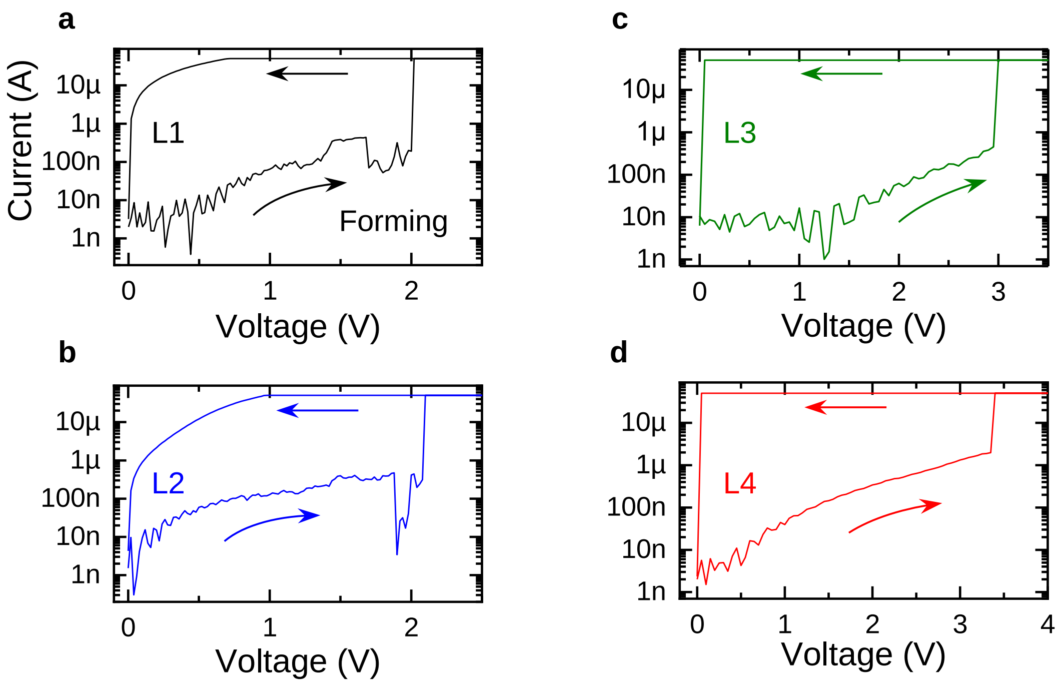

Figure S5. The initial forming process of L1–L4 VRRAMs. a-d Measured *I*–*V* characteristics of the initial forming process, for **a** L1, **b** L2, **c** L3, and **d** L4 VRRAMs, showing the soft breakdown and forming the initial conductive filaments in the originally insulating oxide layer. The forming voltages for all layers of VRRAMs are below 3.5 V.

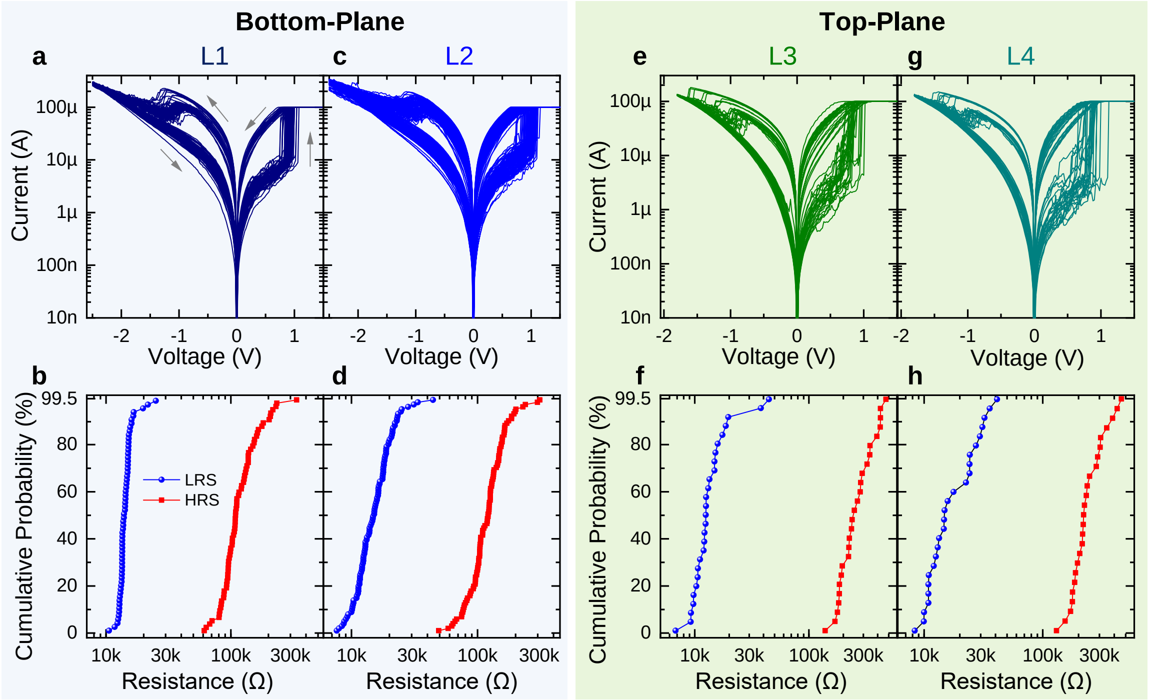

Figure S6. Electrical characterizations for each layer of VRRAM, with V_{GS}=7 V the current limited by the equipment. a–d The DC *I–V* sweeps (top) and the corresponding resistance distributions (bottom) of the bottom-plane two-layer VRRAMs, for **a–b** L1, and **c–d** L2 VRRAMs. The current is limited by the equipment because the MoS_2 transistor can supply a higher current than the equipment compliance current (100 μA), which leads to a lower LRS resistance state compared with those shown in Fig. 2. **e–h** The measurements of the top-plane two-layer VRRAMs shown in the same sequence as in **a–d**, for **e–f** L3, and **g–h** L4 VRRAMs.

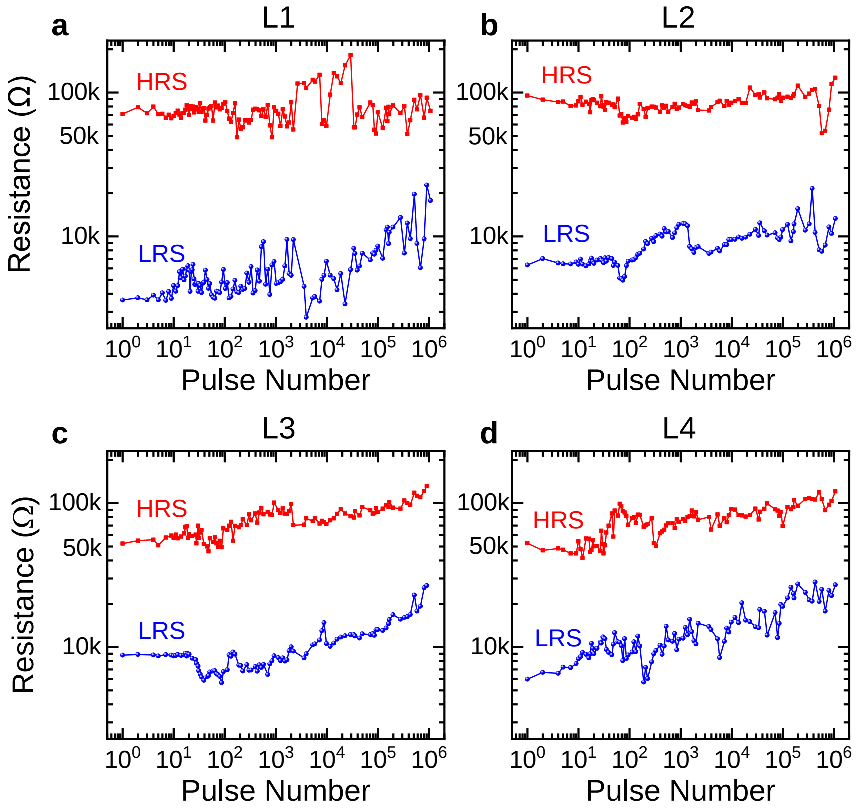

Figure S7. Endurance measurements for all 4 layers of VRRAMs. a–d Measured VRRAM resistances with increasing voltage pulse numbers, using successive pulses of +1.6 V for set operation, and -2.5 V for reset operation, for four layers of VRRAMs, respectively. The devices in all four layers can switch stably for more than 10^6 cycles without any write-verify techniques used.

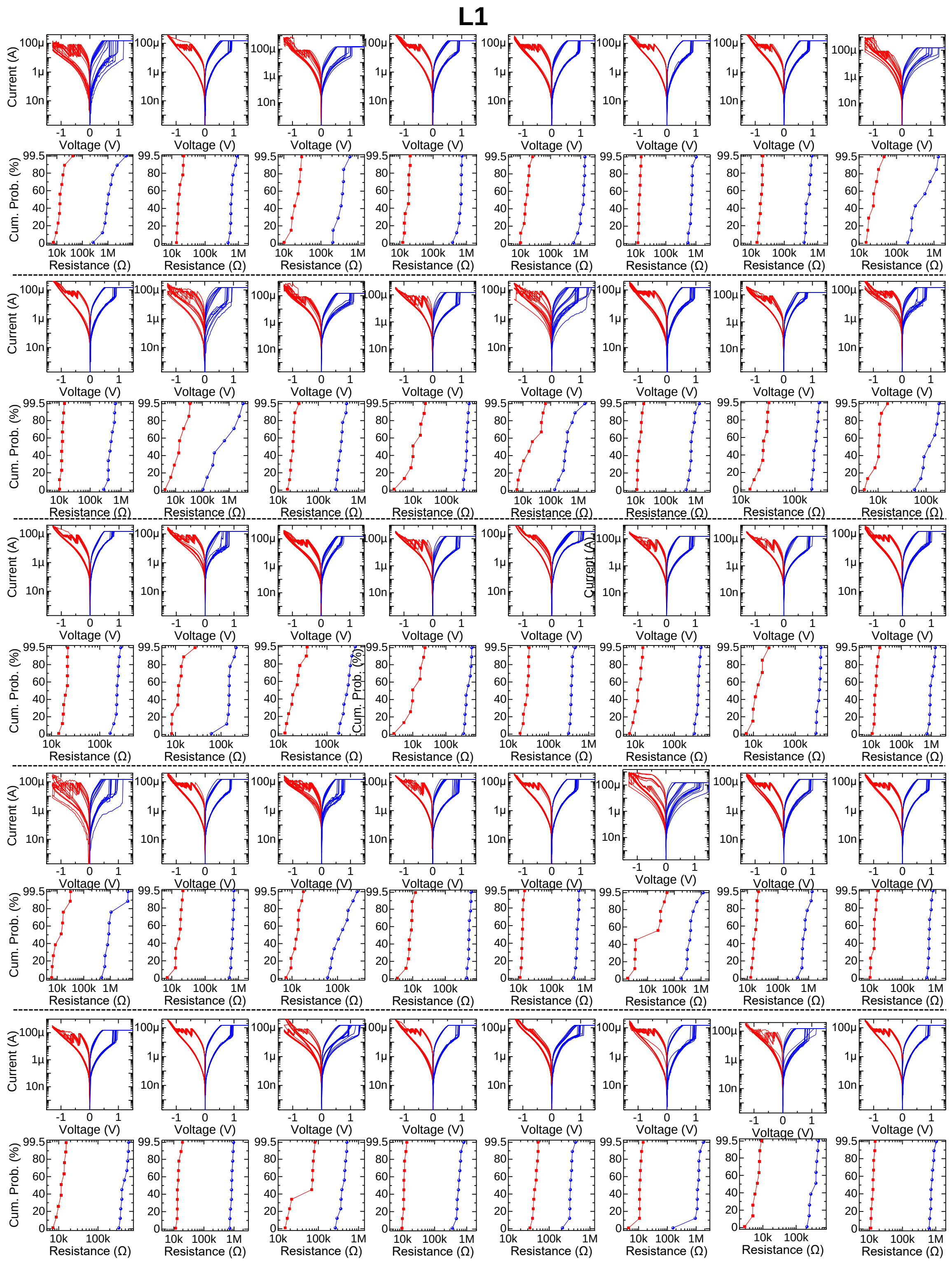

Figure S8. Measured *I-V* characteristics for 40 VRRAMs in Layer 1 (L1), each for multiple cycles, with the corresponding resistance distribution for each device shown below.

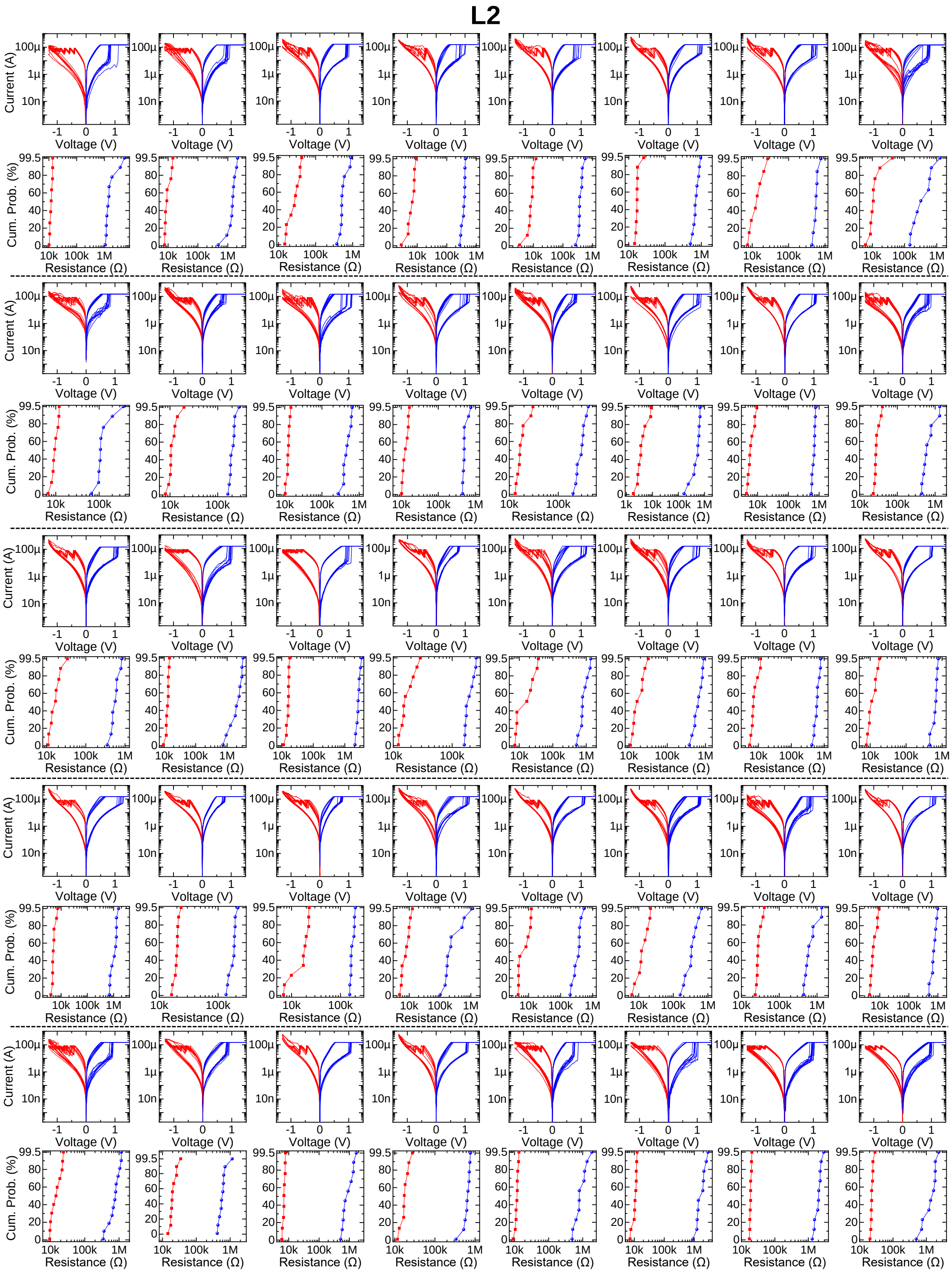

Figure S9. Measured *I-V* characteristics for 40 VRRAMs in Layer 2 (L2), each for multiple cycles, with the corresponding resistance distribution for each device shown below.

L3

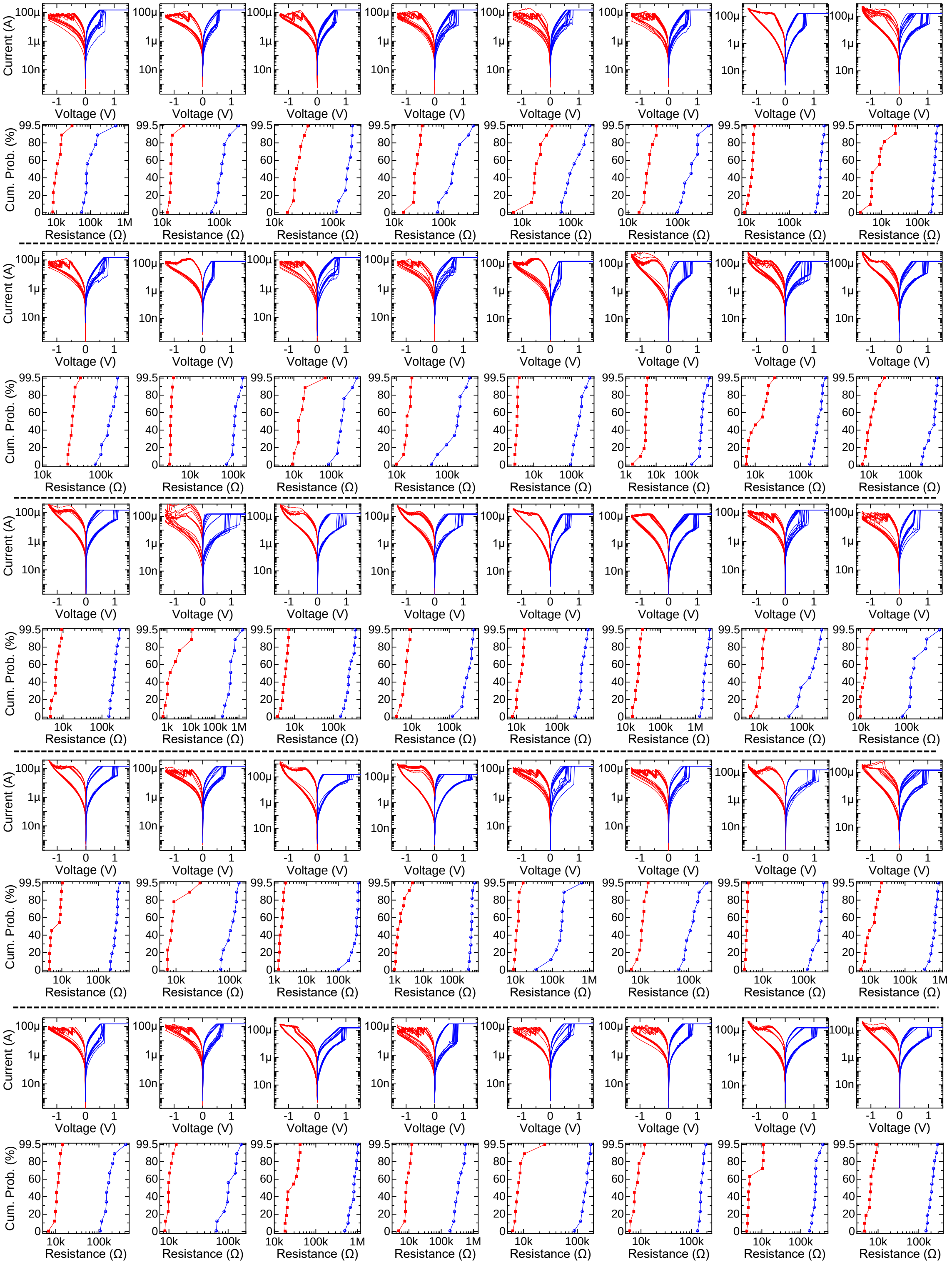

Figure S10. Measured *I-V* characteristics for 40 VRRAMs in Layer 3 (L3), each for multiple cycles, with the corresponding resistance distribution for each device shown below.

L4

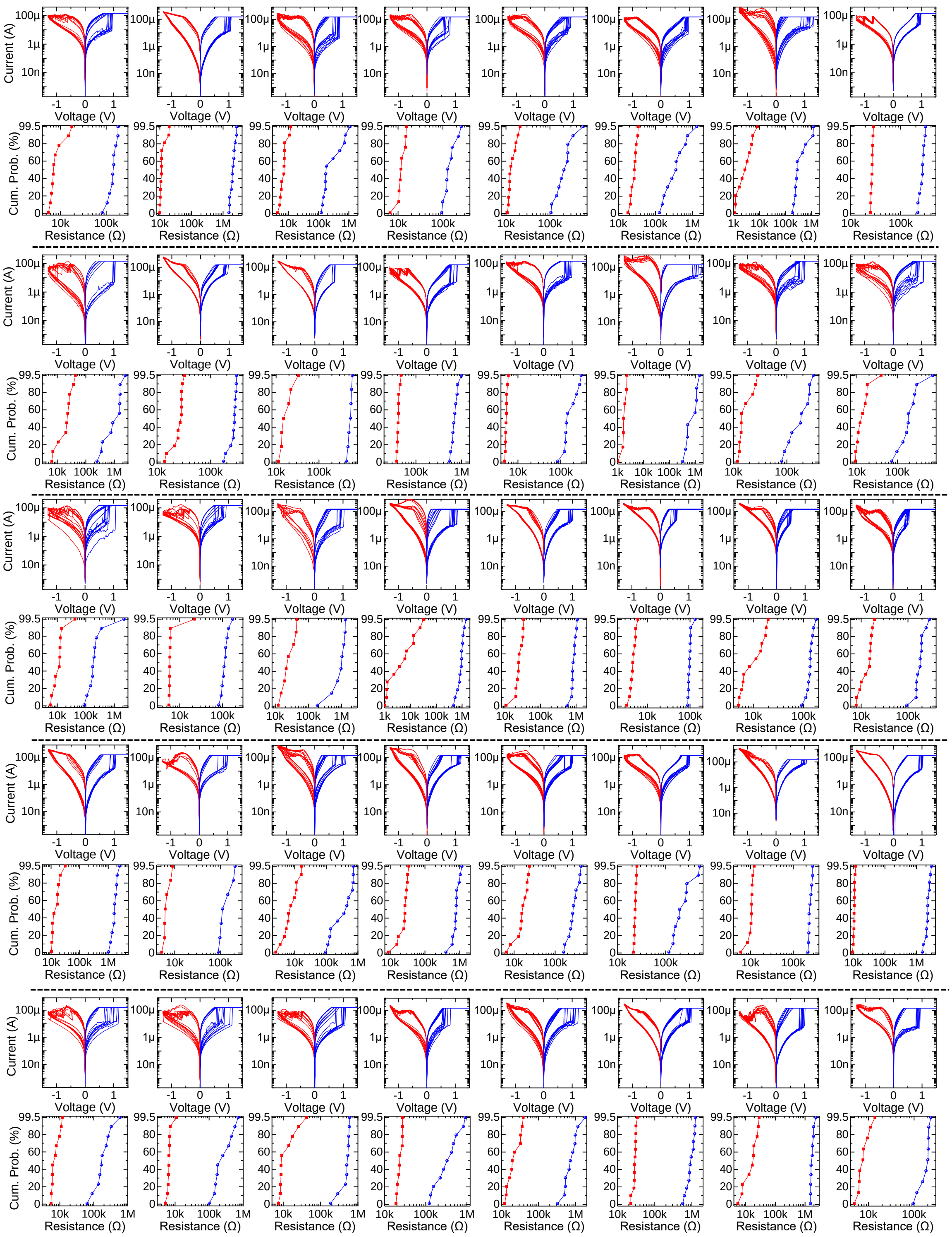

Figure S11. Measured *I-V* characteristics for 40 VRRAMs in Layer 4 (L4), each for multiple cycles, with the corresponding resistance distribution for each device shown below.

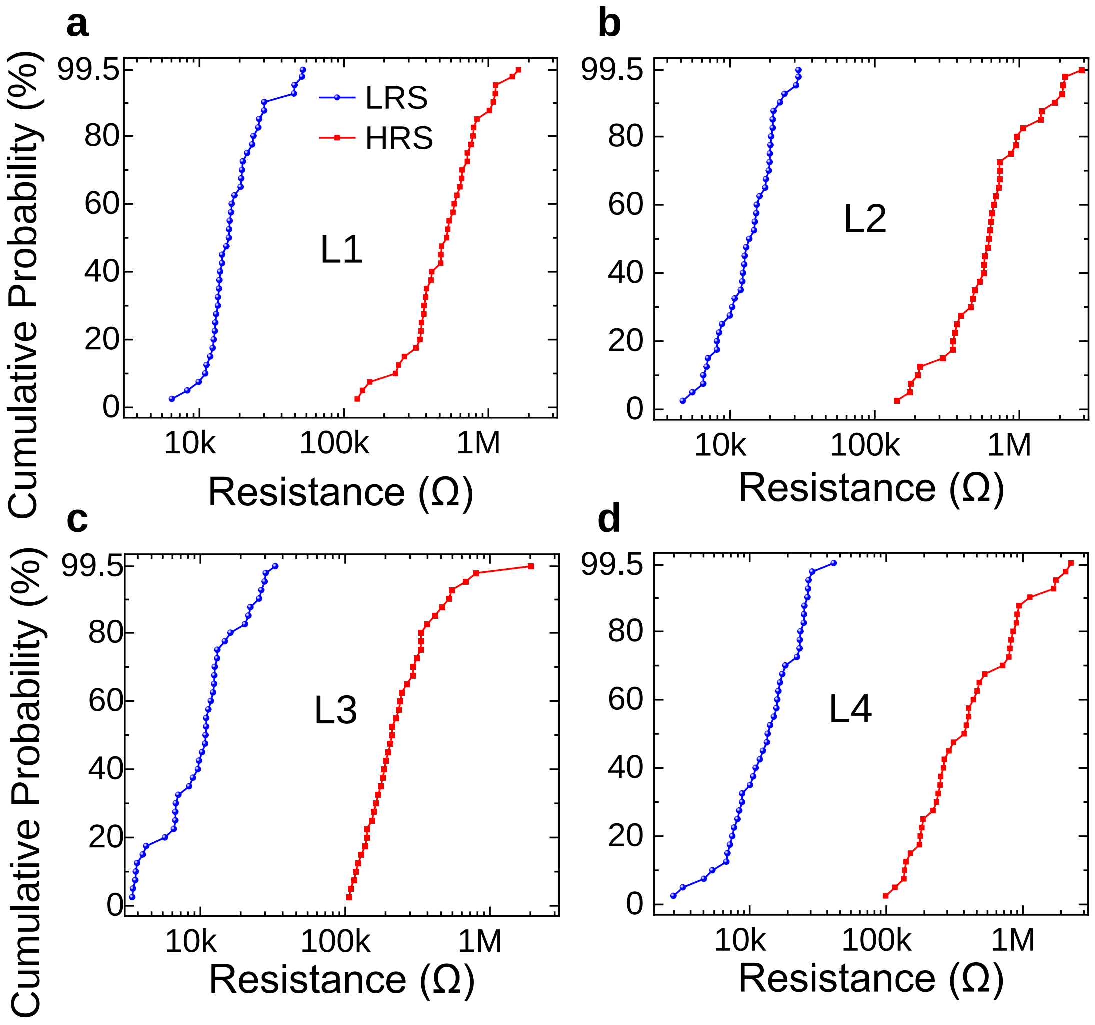

Figure S12. Electrical characterizations for device-to-device variation of four layers of VRRAMs. a–d The resistance distributions for L1–L4 VRRAMs, from the DC *I*–*V* sweeps shown in Fig. S8–S11, respectively, with each layer containing device-to-device variations from 40 VRRAMs. For each device, from the multiple repeated measurement cycles of *I*–*V* sweeps, the mean values of the LRS and HRS resistance states are taken.

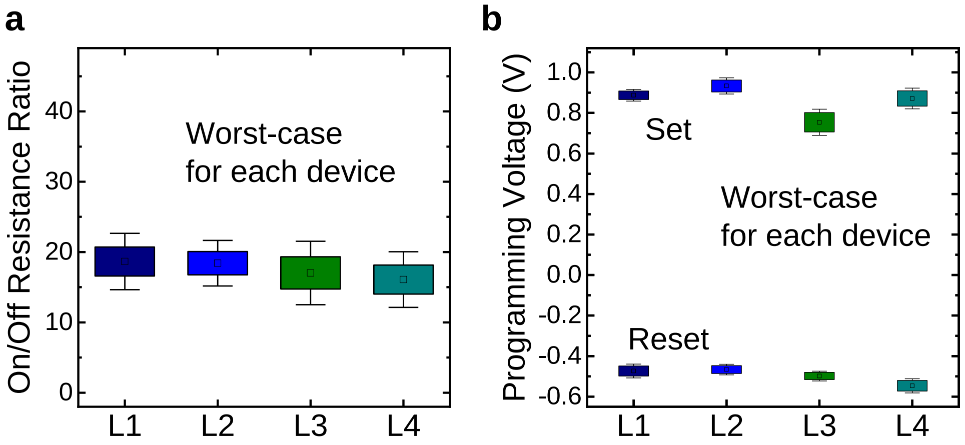

Figure S13. Summary of worst-case device-to-device variation of the 40 VRRAMs shown in Fig. S8–S11. a The variation of the On/Off resistance ratio for L1–L4 VRRAMs, where for each device, the worst-case On/Off resistance ratio during multiple cycles of measurements is used. **b** The variation of the worst-case set and reset voltages for L1–L4 VRRAMs.

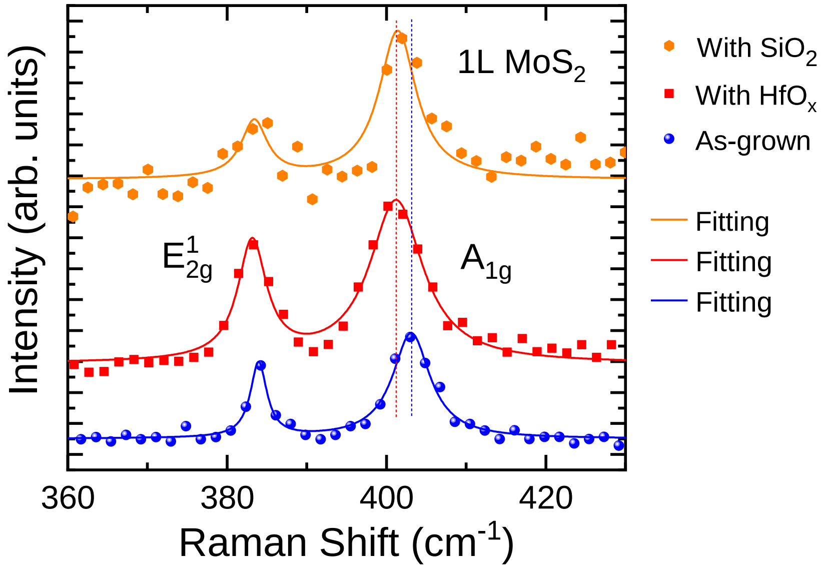

Figure S14. Comparison of Raman spectra before and after the processing, showing the phonon softening effects after the ALD process. The experimental data for as-grown MoS_2, MoS_2 covered with AlO_x and HfO_x layers, and MoS_2 covered with AlO_x, HfO_x, and SiO_2 layers are shown by the blue, red, and orange symbols, respectively, and the fitting to the Raman peaks for as-grown and processed MoS_2 are shown by the blue, red, and orange lines, respectively. The vertical dashed lines show the peak positions of the A_{1g} mode, from 403.1 cm^{-1} before processing to 401.2 cm^{-1} after processing. The data is taken from the Raman mapping in Fig. S15, measured using excitation wavelength of 532 nm.

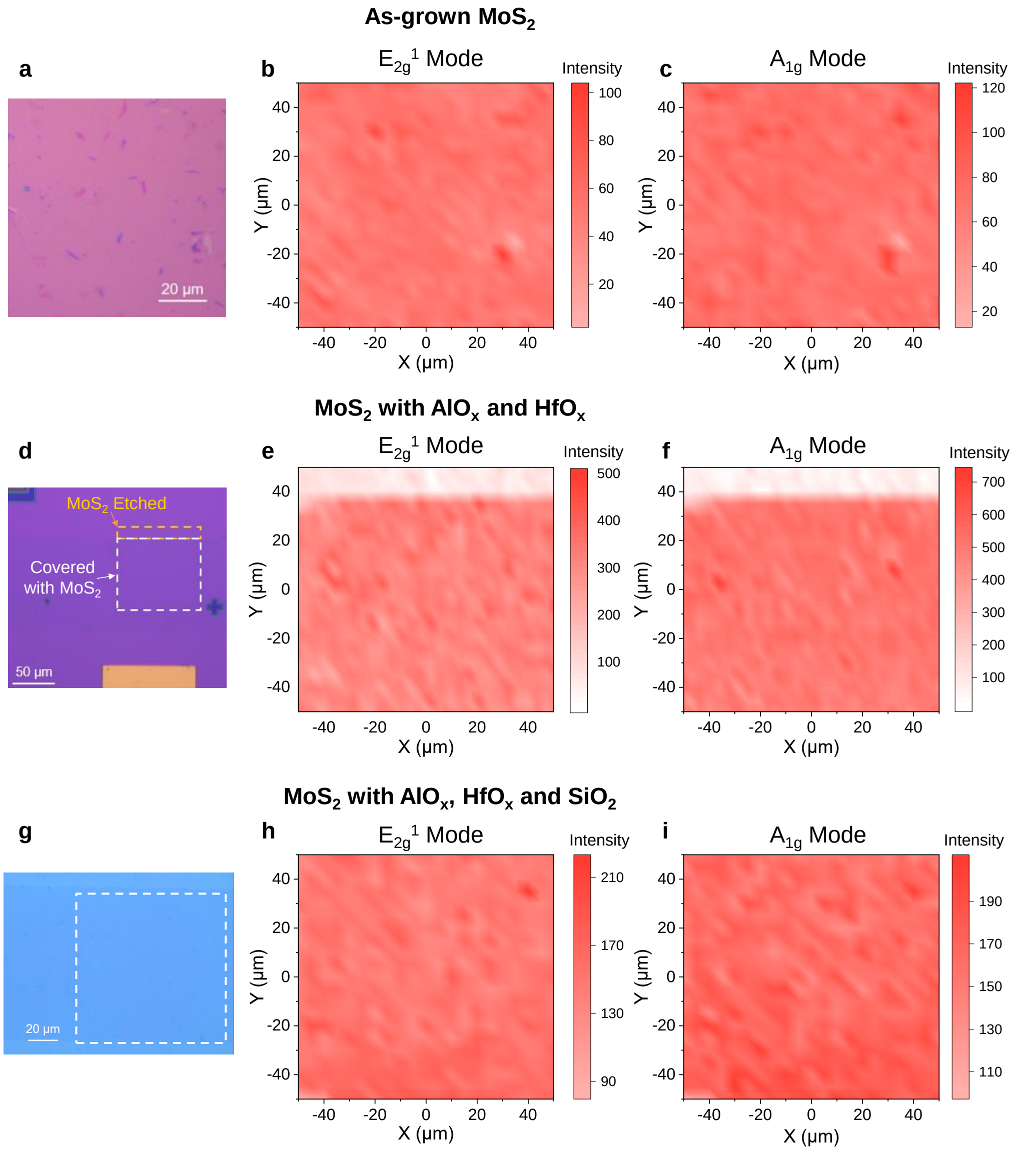

Figure S15. Raman mapping data of MoS_2. a–c Raman mapping of as-grown MoS_2, showing **a** the optical image of the scanned region, **b** the Raman intensity mapping for E_{2g}^1 mode, and **c** the Raman intensity mapping for A_{1g} mode. **d–f** Raman mapping of the processed MoS_2 covered with 1.5 nm AlO_x and 10 nm HfO_x, shown in the same sequence as in **a–c**. The region with MoS_2 etched away is shown by the dashed orange box in **d** and on the top part of **e** and **f**, as a comparison to the region with remaining MoS_2, as shown in the dashed white box in d, and in the bottom part of **e** and **f**. **g–i** Raman mapping of the processed MoS_2 covered with 1.5 nm AlO_x, 10 nm HfO_x, and 100 nm SiO_2, shown in the same sequence as in **a–c**. The dashed box in **g** shows the Raman mapping area.

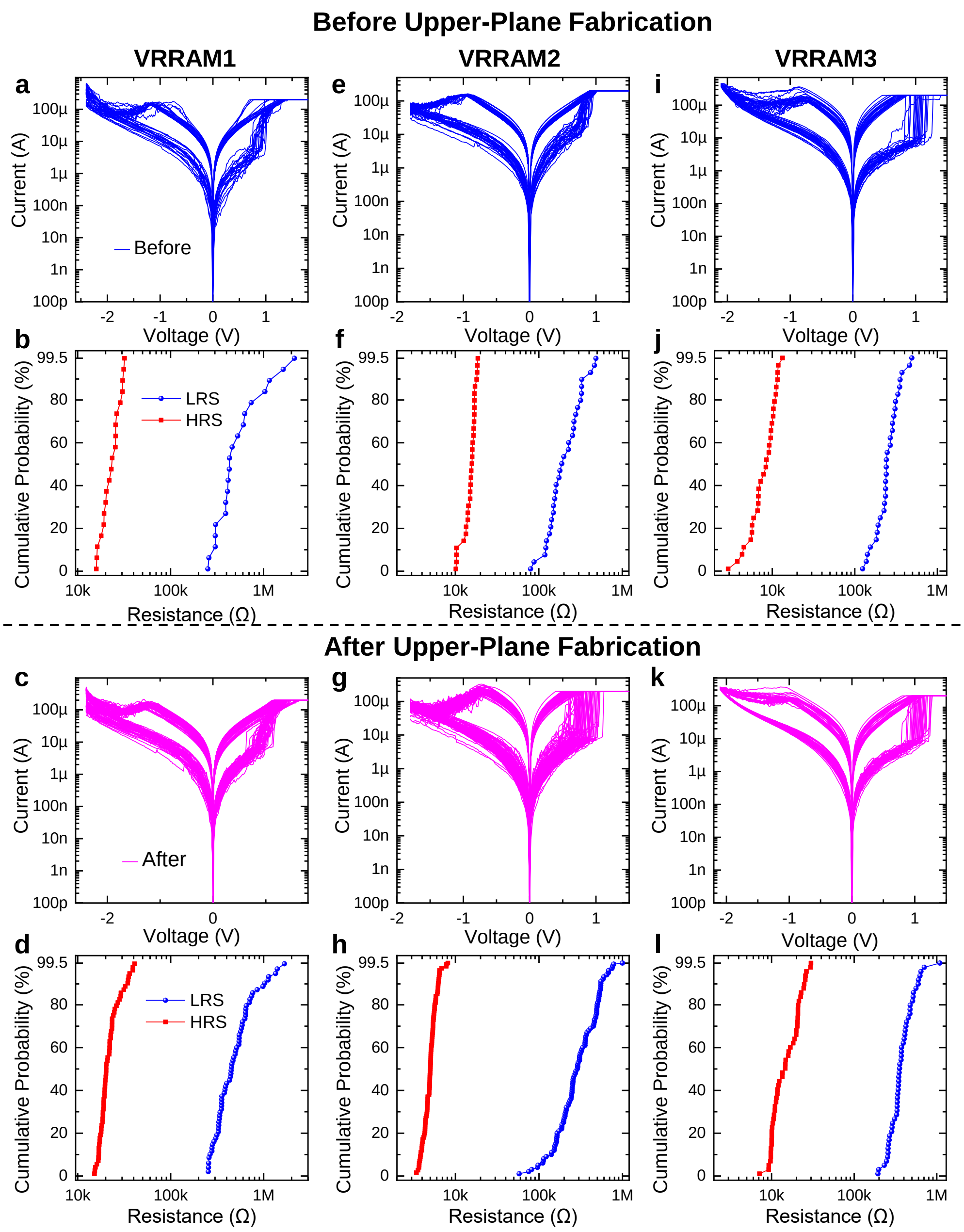

Figure S16. Comparison of the resistive switching characteristics for the same bottom-plane VRRAM device before and after the top-plane device fabrication. a–d Measurements for VRRAM Device 1, showing **a** multiple cycles of resistive switching characteristics, and **b** distribution of the LRS and HRS resistances, before the upper-plane device fabrication. **c–d** Measurements for the same device after the upper-plane device fabrication. **e–h** and **i–l** show measurement results for another two VRRAMs, in the same sequence as **a–d**. The resistive switching *I–V* curves before and after the upper-plane device fabrication are similar for all three VRRAM devices.

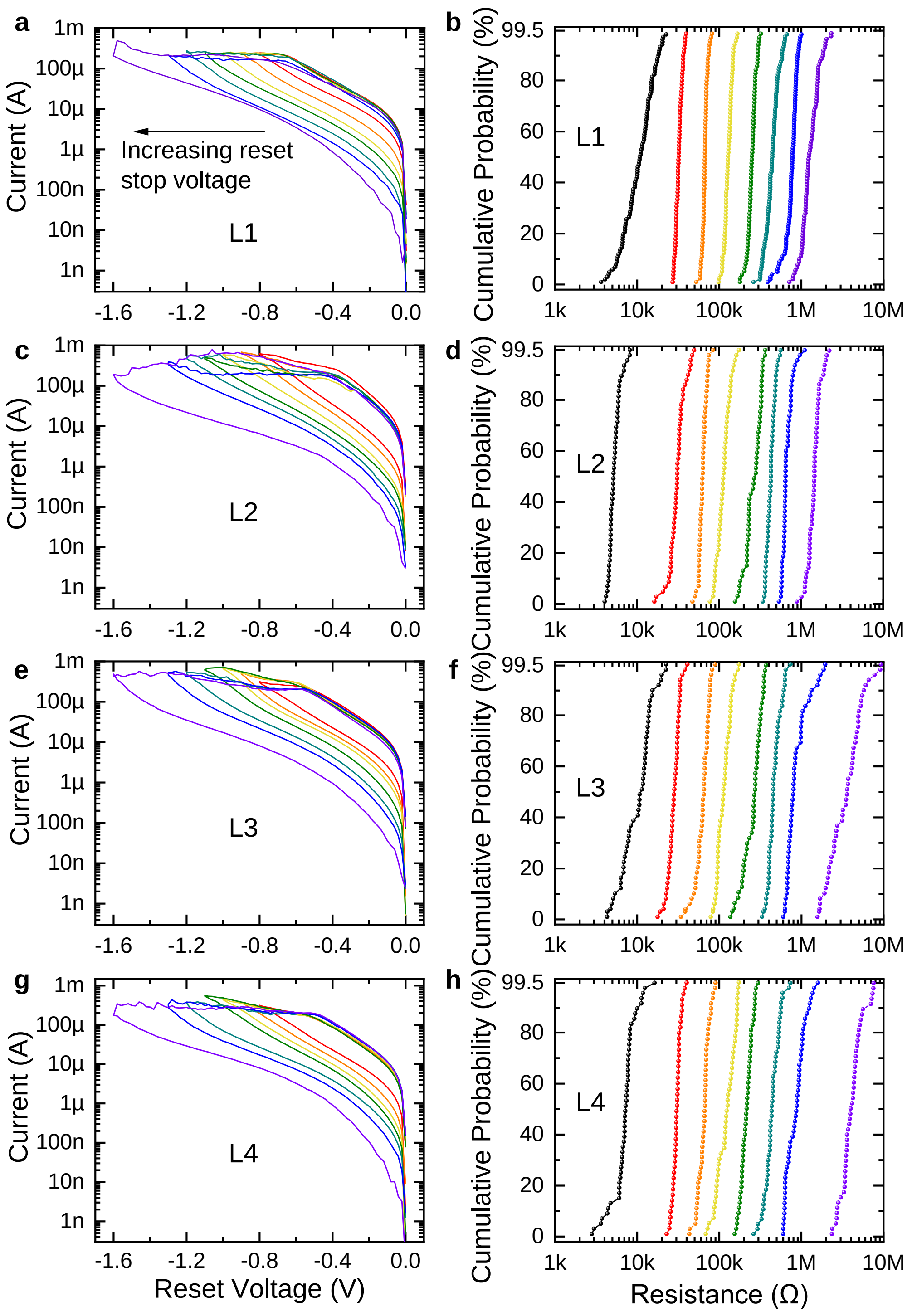

Figure S17. Measurements for multi-level cells by varying reset voltages. a–b Layer 1 VRRAM **a** switching *I–V* curves during reset processes, when using different reset voltages as shown by different colors, and **b** resistance distribution showing 8 stable resistance levels. **c–d, e–f, g–h** show the multi-level cell measurements for Layer 2, Layer 3, and Layer 4 VRRAMs, respectively.

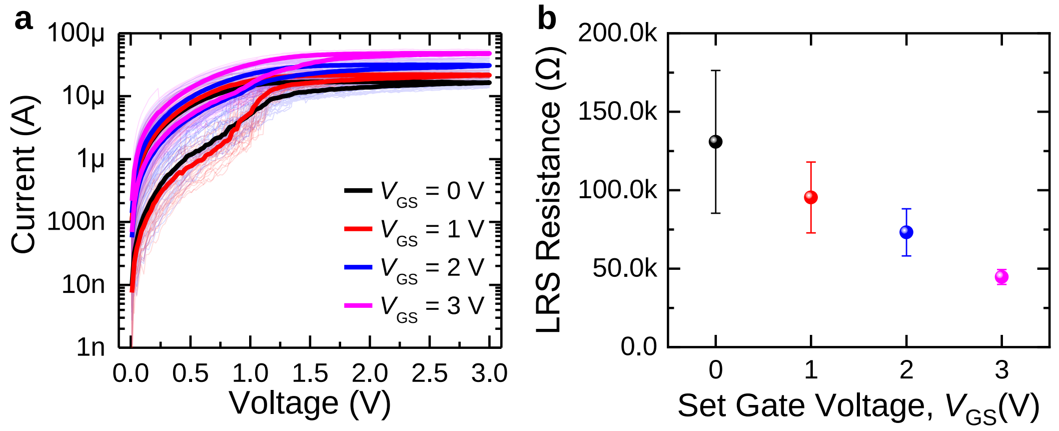

Figure S18. The effect of MoS_2 FET gate voltage on memory cell resistance states during the set process, which provides another technique to achieve multi-level cells. a *I-V* characteristics during the set process for 30 switching cycles (transparent lines) and their averaged curve (highlighted lines), at different gate voltages. **b** Summary of LRS resistances at different gate voltages during set operation, showing that a larger gate voltage allows a larger maximum current through the transistor and thus a larger filament size, leading to a smaller LRS resistance.

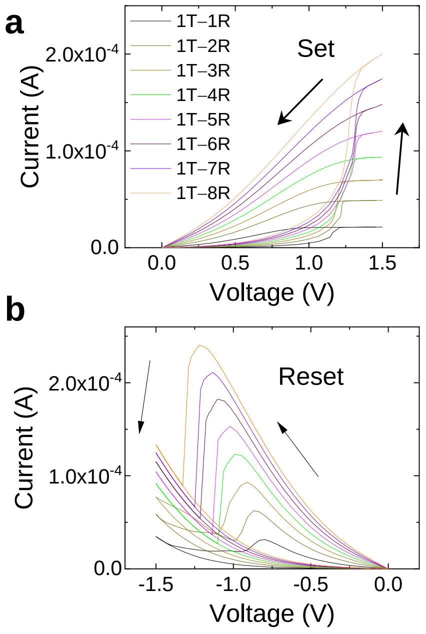

c

Set	V_{GS}	V_{DS}	V_{BE}
1T–1R	0.2 V	1.5 V	GND
1T–2R	0.6 V	1.5 V	GND
1T–3R	1.1 V	1.5 V	GND
1T–4R	1.7 V	1.5 V	GND
1T–5R	2.3 V	1.5 V	GND
1T–6R	3 V	1.5 V	GND
1T–7R	3.6 V	1.5V	GND
1T–8R	4.3 V	1.5 V	GND
1T–9R	5 V	1.5 V	GND
1T–10R	5.6 V	1.5 V	GND
1T–11R	6.4 V	1.5 V	GND
1T–12R	7 V	1.5 V	GND
Reset	8 V	GND	1.5 V
Read	7 V	0.1 V	GND

Figure S19. Simulation parameters for driving current simulation. a-b Simulated parallel switching currents for 1T–1R to 1T–8R schemes during the **a** set process, and **b** reset process, using HSPICE based on the fitted RRAM and FET models. **c** The operation modes for different 1T–*n*R schemes, for Fig. 5f and Fig. 5g in the Main Text. The gate voltages (V_{GS}) during the set process are carefully selected, to make sure the FETs can provide enough parallel switching current, because as the number of parallel switching VRRAMs increases, the needed switching current for both set and reset processes will increase. At the same time, the gate voltages are also chosen to be not too large, to avoid a large compliance current that can lead to severely small LRS resistance. The voltage at the bottom electrode of the VRRAMs (V_{BE}) remains unchanged.

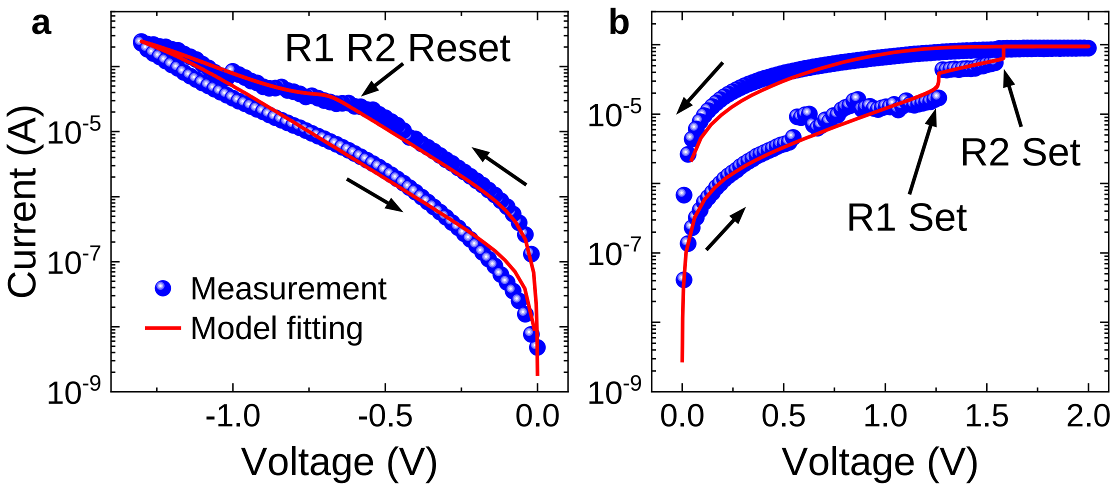

Figure S20. Parallel reset and set capability. a–b Measured parallel **a** reset and **b** set behaviors for the 1T–4R structure, with the total current shown by the blue symbols, when two of the VRRAM layers are programmed together. The red lines show the fitting to the parallel reset and set process using HSPICE. In the parallel set behavior, due to variation in set voltages for two layers of VRRAMs, two sudden increases of current are observed, corresponding to the set behavior for each layer of VRRAM, respectively.

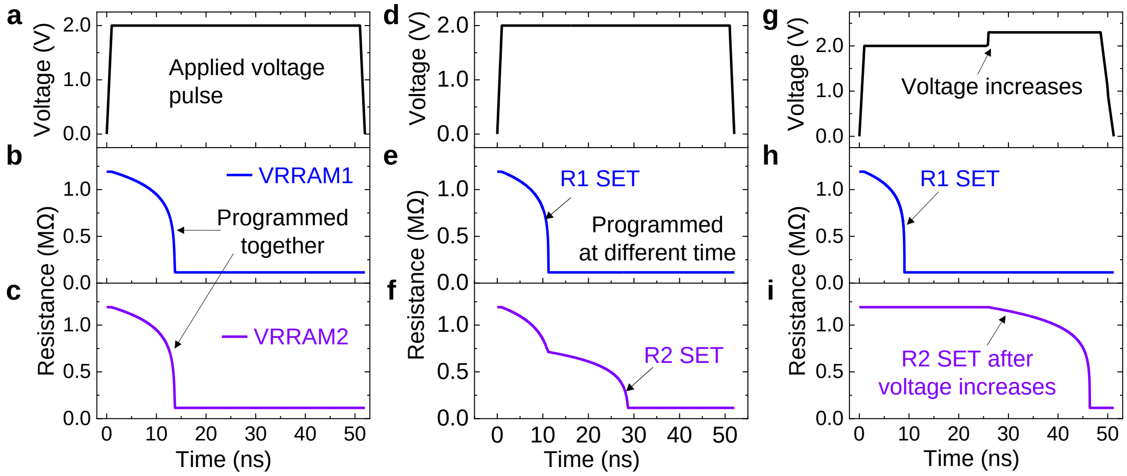

Figure S21. Simulation about the effect of device variation on parallel set behavior using HSPICE. a–c Top to bottom figures show the applied set voltage, and the resistances of VRRAM1 and VRRAM2 as a function of time, respectively, during the parallel set operation. It shows that the two layers of VRRAMs are programmed simultaneously with identical switching behaviors. **d–f** The simulation results shown in the same sequence as in **a–c**, when the ON-state resistance of the MoS_2 transistor is much smaller than the LRS resistance of the VRRAM. Device variation (non-ideality) is already taken into consideration. Both VRRAMs are programmed with the same voltage pulse amplitude, but they are fully switched at different time. **g–i** The simulation results shown in the same sequence as in **a–c**, when the ON-state resistance of the MoS_2 transistor is similar to the LRS resistance of the VRRAM, and the worst-case variation in set voltage is taken into consideration. After VRRAM1 is programmed first, a boosting of set voltage amplitude is needed to program VRRAM2.

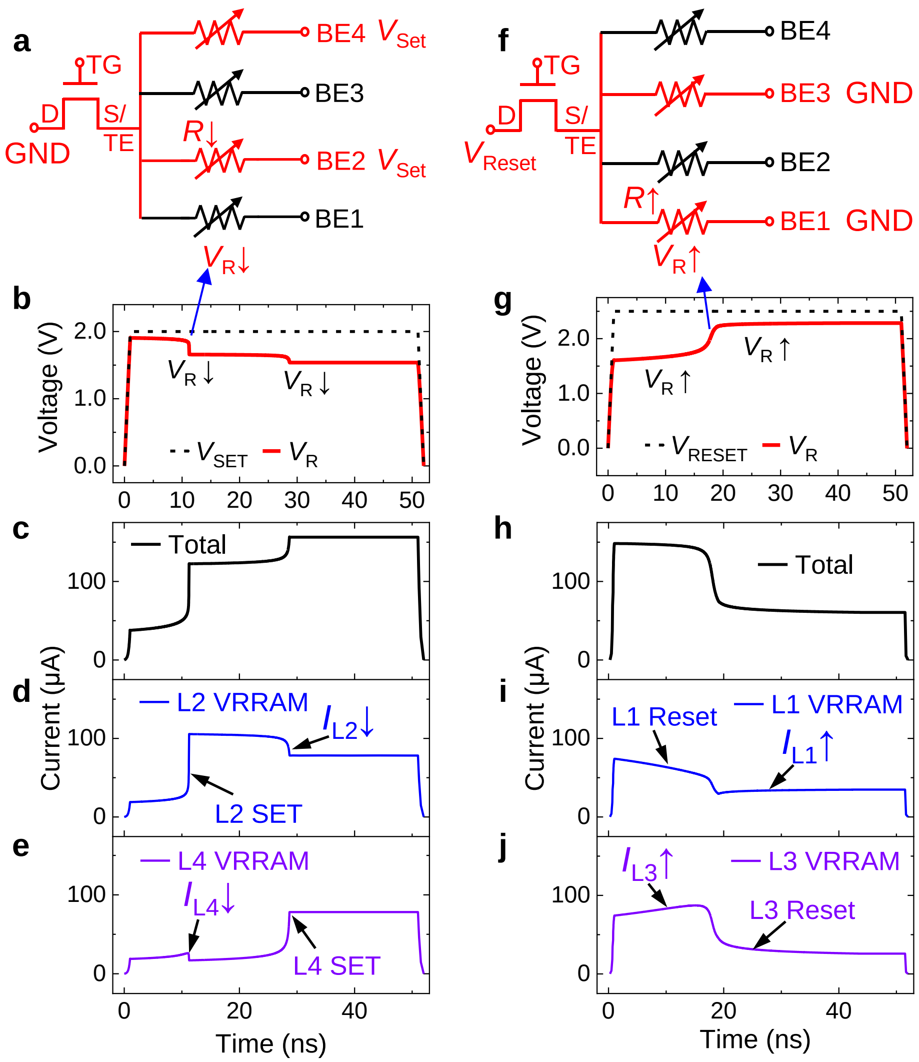

Figure S22. Simulation and comparison about the effect of device variation on parallel set and reset behaviors using HSPICE. a–e Simulation of the parallel set process using the parameters in Fig. S21 d-f, which requires a longer voltage pulse duration but no boosting of voltage amplitude. **a** Circuit schematic of the 1T–4R structure with two layers of VRRAMs (L2 and L4) set in parallel, showing that the voltage V_R on 2 layers of parallel VRRAMs drops when L2 VRRAM is set first, due to the voltage division with the series transistor, making the set of L4 VRRAM more challenging. **b** The voltage drop on the two parallel VRRAMs. **c–e** The total current, current across L2 VRRAM, and current across L4 VRRAM, respectively. **f–j** Simulation of the parallel reset process, shown in the same sequence as in **a–e**, showing that if L1 VRRAM is reset first, the voltage across the VRRAM increases, making the reset of L3 VRRAM easier.

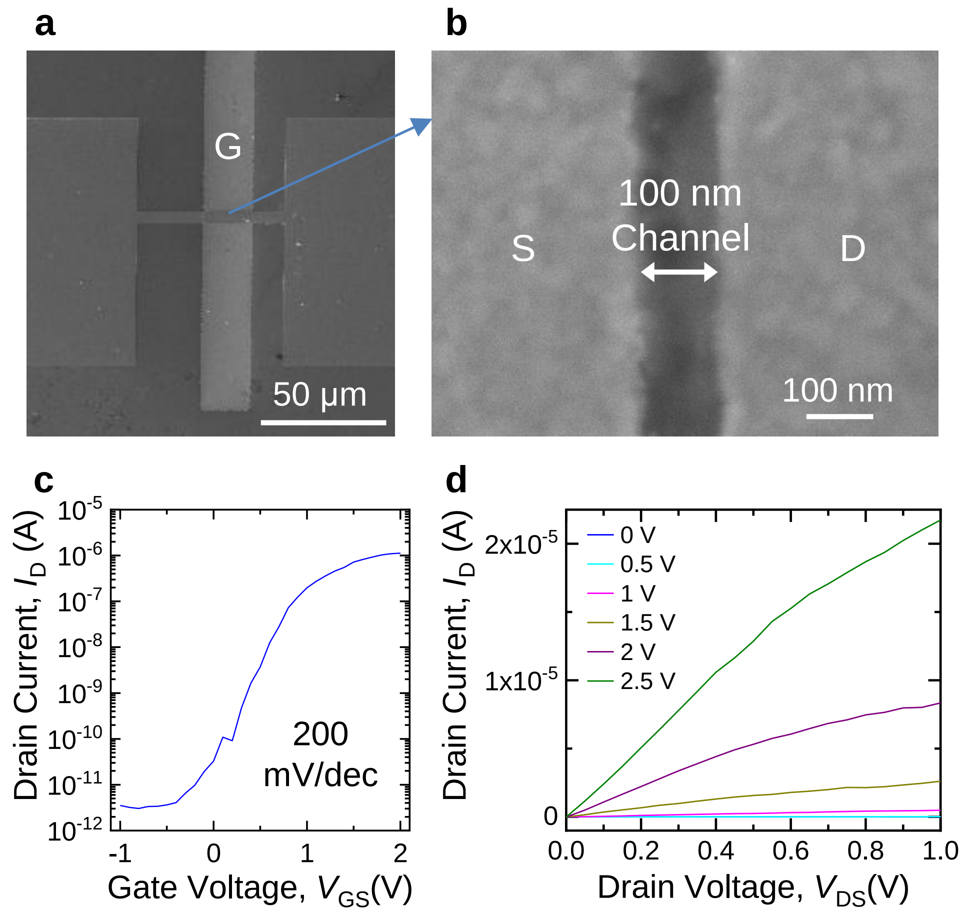

Figure S23. Measured switching behavior of 2D MoS_2 transistors with 100-nm channel length. **a**–**b** Scanning electron microscopy (SEM) images of a 2D MoS_2 transistor with 100-nm channel length and *W*/*L* of 100, with local back gates, and HfO_x gate dielectric, showing the **a** zoom-out and **b** zoom-in images. **c**–**d** Measured **c** I_D-V_{GS} characteristic at V_{DS} = 0.1 V, and **d** I_D-V_{DS} curves when V_{GS} varies from 0 V to 2.5 V. The device shows a subthreshold swing of 200 mV/dec. The gate and drain voltages remain relatively low to prevent leakage.

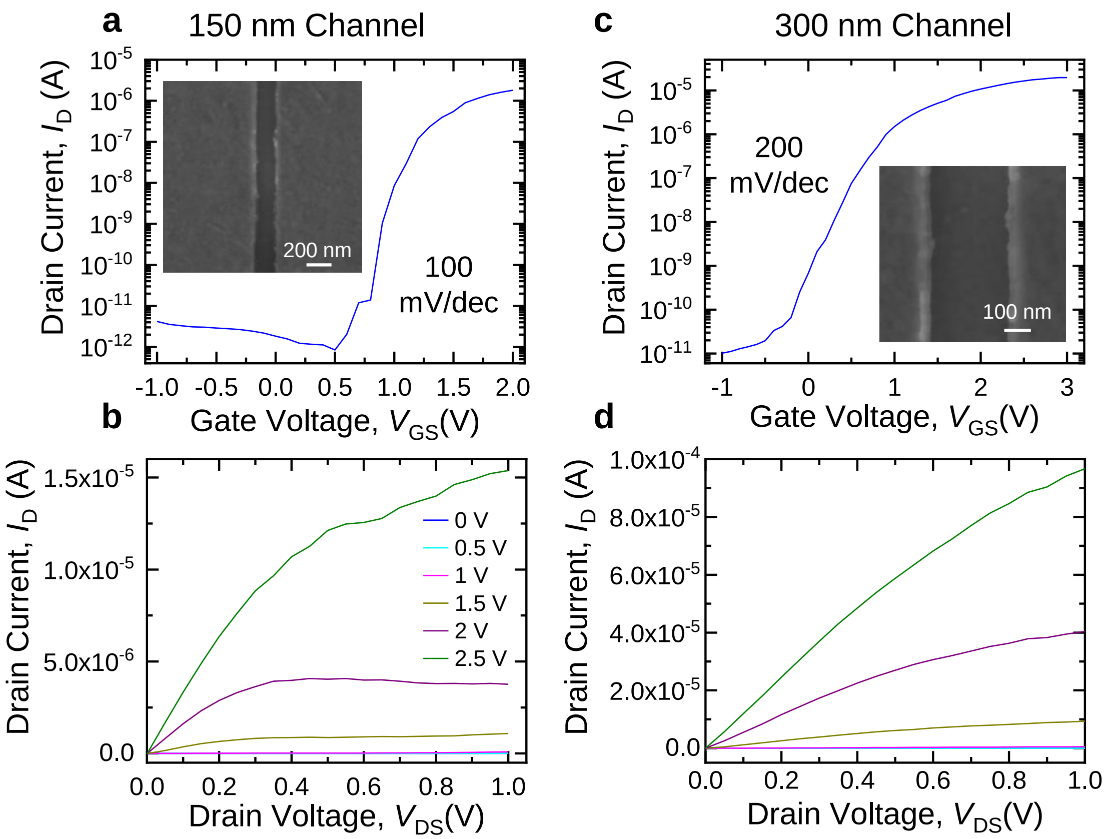

Figure S24. Measured switching behavior for 2D MoS_2 transistors with 150 nm and 300 nm channel lengths. a–b Measured **a** I_D-V_{GS} characteristic at V_{DS} = 0.1 V, and **b** I_D-V_{DS} curves when V_{GS} varies from 0 V to 2.5 V, for a MoS_2 transistor with channel length of 150 nm and W/L of 50. The device shows subthreshold swing of 100 mV/dec. *Inset*: SEM image. **c–d** Measured **c** I_D-V_{GS} and **d** I_D-V_{DS} curves for a MoS_2 transistor with channel length of 300 nm and W/L of 100. The device shows subthreshold swing of 200 mV/dec. *Inset*: SEM image.

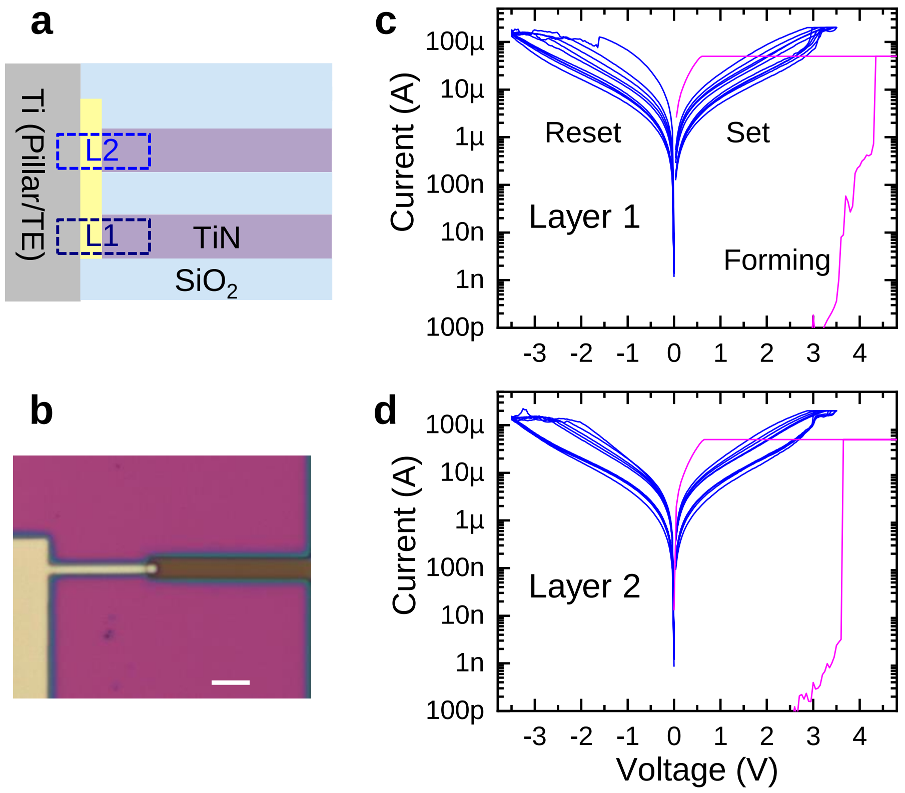

Figure S25. VRRAM based on TiN/HfO$_x$/Ti structure for CMOS compatibility. a Cross-sectional illustration of the two-layer VRRAMs. **b** Optical image of a VRRAM device. *Scale bar*: 10 μm. **c–d** Measurement results for the switching characteristics of the VRRAMs, showing the forming, and multiple cycles of set and reset processes, for **a** Layer 1, and **b** Layer 2 VRRAMs.

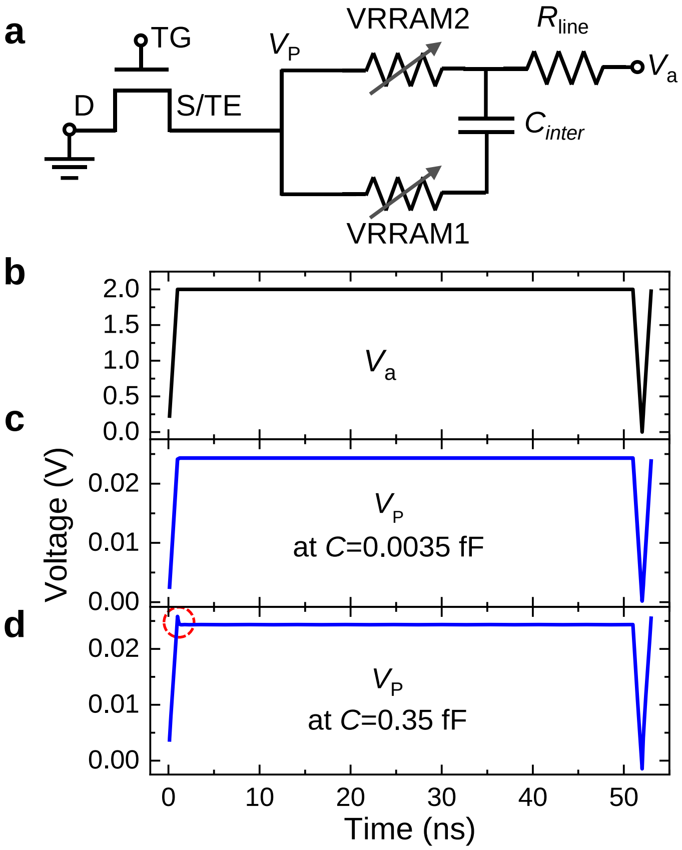

Figure S26. **Simulation of the interlayer capacitance effect on latency using HSPICE. a** Schematic of the equivalent circuit of a 1T–2R structure, including the parasitic interlayer capacitance C_{inter} and resistance R_{wire} of the metal interconnect wires. **b–d** The simulated timing sequence diagram, showing **b** the applied voltage V_{a}, and **c** the voltage response at V_{P} as shown in **a**, with metal wire's overlapping area of 100 nm×100 nm, and dielectric thickness of 100 nm, which corresponds to the capacitance of C=0.0035 fF. The response shows almost no delay in V_{P}. **d** The voltage response at V_{P} as shown in **a**, with overlapping area increased to 1 μm×1 μm, which corresponds to the capacitance of C=0.35 fF. As shown in the dashed red circle in **d**, the delay in this case is less than 0.3 ns. In the simulation, the metal wire sheet resistance $R_{\square}$ is assumed to be 0.175 Ω/□, and the wire length is 10 μm.

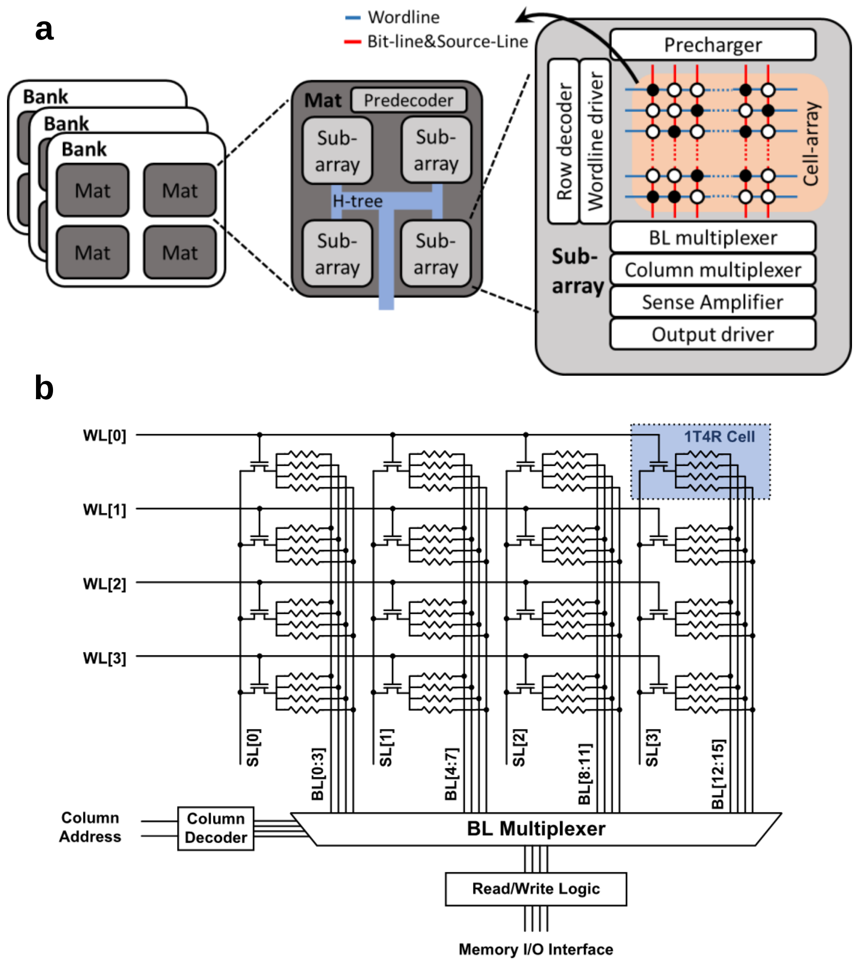

Figure S27. **The structure for circuit simulation. a** Memory organization adopted in this work, by modification from Ref. [24]. **b** Illustration of an example memory array with 4×4 1T–4R VRRAM cells.

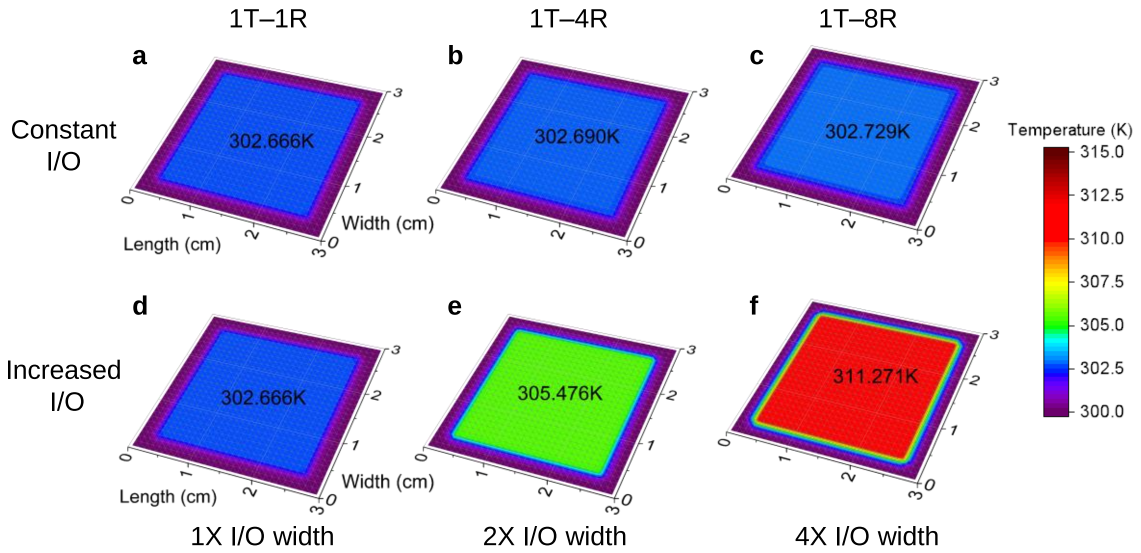

Figure S28. **Thermal simulation for the 1T–*n*R structure during continuous write operations applied to the bottom VRRAM layer, which is furthest from the heat sink at the top and shows the worst case scenario, with comparison to the 1T–1R structure. a–c** With the fixed I/O width, the simulated temperature map on the bottom VRRAM layer (the layer furthest away from the heat sink), for **a** 1T–1R, **b** 1T–4R, and **c** 1T–8R structures, respectively. The highest temperature is noted in the middle, showing that there is no significant increase of the temperature inside the system. **d–f** With our improved memory density, we increase the I/O width for our 1T–*n*R structure, to explore the upper bound of thermal dissipation problem. The temperature map is simulated with an initial 256 I/O for **d** 1T–1R structure, 512 I/O for **e** 1T–4R structure, and 1024 I/O for **f** 1T–8R structure. Note that with the increased I/O width, the highest temperature of the 1T–8R structure only increases by less than 12K. The heat transfer coefficient of the heat sink is set as 1.0×10^{-7} W/(μm^2·K), and the environment temperature is set as 300K.

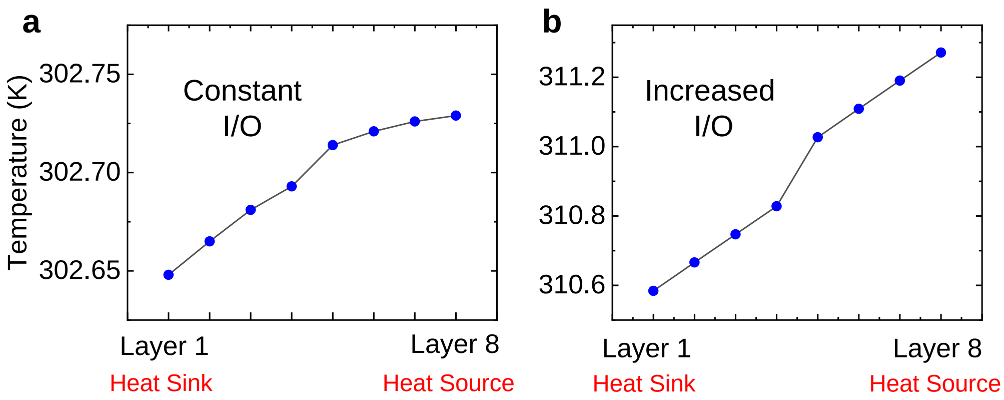

Figure S29. **The maximum temperature for each device layer in 1T–8R structure.** Maximum temperature for **a** fixed I/O width, and **b** Increased I/O width by *n*/2 times, where *n* is the number of VRRAM layers. The temperature in the vertical structure gradually decreases from Layer 8 (bottom, heat source) to Layer 1 (top, heat sink).

Supplementary Tables

Table S1. Fitting parameters for transistors and VRRAMs. a The main fitting parameters of the 130 nm BSIM4 Predictive Technology Model (PTM)[1]. **b** The detailed fitting parameters of the RRAM compact model[2].

a

Parameter	Value
V_{th0}	-0.3782
T_{ox}	1.125×10^{-8}
X_j	3.92×10^{-8}
V_{sat}	1.13×10^{6}
V_{off}	-0.13
N_{factor}	1.5
C_j	0.0005
U_0	0.05928
U_a	6×10^{-10}
U_b	1.2×10^{-18}
U_c	0

b

Parameter	Unit	Value	
		Set	Reset
g_0	nm	0.25	0.25
g_1	nm	1	1.2
V_0	V	0.235	0.235
v_0	m/s	10	10
I_0	A	0.0001	0.0001
α	1	3	1
β	1	0.7	12
γ_0	1	15	30
E_a	eV	0.8	1
δ_{g0}	nm	0.012	0.012
g_{min}	nm	1.45	1.45
g_{max}	nm	2.9	2.9

Table S2. The detailed parameters used in the circuit-level modeling *via* NVSim. The parameters are extracted from the measurement data. During the simulation, the parameters of the transistors and VRRAMs are unchanged while sweeping the memory capacity from 8 MB to 64 MB, for 1T–1R, 1T–4R, and 1T–8R schemes.

Technology Node (nm)	65	LRS (Ω)	10K
Capacity (MB)	8, 16, 32, 64	HRS (Ω)	300K
Word Length (Bit)	256	V_{set} (V)	+1.1
Array Size	1024×1024	V_{reset} (V)	−1.4
Cell Area (F^2)	24	Pulse Width (ns)	60
ADC, Mux	16:1	Rise/Fall Time (ns)	20
1T Width (F)	4	E_{set} (pJ)	1.3
1T Current (μA)	>100	E_{reset} (pJ)	3.4

Table S3. The simulation results for 64MB of 1T–1R, 1T–4R, and 1T–8R memory array *via* NVSim. The 1T–*n*R structure will bring significant benefit compared with the 1T–1R structure, which is mainly attributed to the reduction of data transfer length due to the smaller lateral area when vertical stacking is used.

	Area (mm^2)	Read Delay (ns)	Write Delay (ns)	Read Energy (pJ)	Write Energy (nJ)
1T–1R	47.792	4.801	52.506	1233	3.100
1T–4R	12.043	2.056	51.131	592.040	2.465
1T–8R	6.069	1.410	50.805	335.913	2.213

Table S4. Comparison among monolithic 3D integration structures.

Structure	Fabrication ≤ 300°C	FET Channel Thickness	Memory Layers Per Unit Area	Total Device Layers (Bottom to Top)	Set Power	Reset Power	Potential for Further Stacking
2T–2R [3]	No	2 nm (CNT) & Bulk (Si)	2	4 (Si FET, Two RRAM layers, CNT FET)	150 μW	300 μW	High
1T–2R [4]	No	Bulk (Si)	1	2 (Si FET, RRAM)	180 nW	40 μW	Low
1T–1R [5]	No	2 nm (CNT) & Bulk (Si)	1	4 (Si FET, CNT FET, RRAM, CNT FET)	50 μW	2 mW	High
2T–2R [6]	Yes	0.65 nm (1L MoS_2)	1	2 (MoS_2 FET, RRAM)	Min: 130 μW<br>Typical: 420 μW	Min: 100 μW<br>Typical: 520 μW	High
2T–2R [7]	Yes	2 nm (CNT)	1	2 (CNT FET, RRAM)	400 μW	400 μW	High
1T–4R [8]	No	Bulk (Si)	2	3 (Si FET, RRAMs, RRAMs)	N.A.	N.A.	Low
1T–1R [9]	Yes	3 nm (WSe_2)	1	1 (3D shown by simulation)	1.6 μW	20 μW	High
2T–1R [10]	No	11 nm (MoS_2), Bulk (Si)	1	2 (Si FET, MoS_2 FET, MoS_2 RRAM)	35 μW	120 μW	High
3T–3R [11]	No	2 nm (CNT) & Bulk (Si)	2	3 (Si FET, $HfAlO_x$ RRAM, CNT FET, and Ta_2O_5 RRAM)	66 μW	45 μW	High
1T–4R [12]	No	Bulk (Si)	4	5 (Si FET, Four-layer VRRAMs)	80 μW	80 μW	High
1T-8R [13]	No	Bulk (Si)	4	5 (Si FET, Four-layer VRRAMs)	N.A.	N.A.	High
3R [14]	Yes	N.A.	3	3 (Three layers of RRAMs)	1 mW	1 mW	High
1T–4R (This Work)	Yes	0.65 nm (1L MoS_2)	4	5 (Two-layer VRRAMs, 2D FET, Two-layer VRRAMs)	Min: 20 μW<br>Typical: 120 μW	Min: 30 μW<br>Typical: 200 μW	Very High

Table S5. Comparison among different memory and storage technologies.

Memory Technology	On-Chip?	Monolithic 3D Stackable	Volatile or Nonvolatile	Write Speed	Memory Density
SRAM	Yes	No	Volatile	~1 ns	Low
DRAM	No	No	Volatile (Requires millisecond refresh)	~10 ns	High
3D NAND	No	No	Nonvolatile	~1 μs	Very High
3D VRRAM (This Work)	Yes	Yes	Nonvolatile	~60 ns	Very High

Table S6. Comparison among different 3D VRRAMs.

VRRAM Layers	2 [15]	2 [16]	2 [17]	2 [18]	3 [19]	2 [20]	2 [21]	4 [12]	4 [22]	8 [23]	4 (This Work)
Device Structure	TiN/ $TiNO_x$ / WO_x /W	TiN/TiON/ HfO_x /Pt	Ta/ TaO_x / TiO_2 /Ti	TiN/ HfO_x /Pt	Pt/ AlO_δ / Ta_2O_{5-x} / TaO_y /Pt	TiN/ HfO_x /Gra-phene	Ta/ TaO_x /Pt	TiN/Ti/ HfO_x /TiN	TiN/ TiO_x / HfO_x /Ru	TiN/ HfO_2 / TaO_x /Ti	Ti/TiN/ HfO_x /Pt
Scalabil-ity (Sim-ulation)	N.A.	10 Mb	10 Mb	N.A.	1 K–10 K bits	200 Kb	N.A.	4 Kb	1 Kb (Exp.)	2 Kb (Exp.)	512 Mb
Switch-ing Layer Size in Cross Section	~ 10 nm × 17 nm	20 nm × 5 nm	60 nm × 100 nm	5 nm × 5 nm	~ 20 nm × 40 nm	5 nm × 0.3 nm	~ 50 nm × 11 nm	20 nm × 5 nm	~ 75 nm × 15 nm	~ 20 nm × 13 nm	33 nm × 5 nm
On/Off	> 10	> 10	10	> 10	$> 10^3$	70	> 10	> 10	$> 10^3$	10^2	10^2
Endur-ance	10^3	10^8	10^{10}	5×10^9	10^{10}	1600	10^8	10^6	10^7	10^7	10^6
Read Latency	N.A.	N.A.	N.A.	N.A.	N.A.	N.A.	N.A.	N.A.	N.A.	300 ns	N.A.
Program Latency	50 ns	50 ns	1 µs	400 ns	100 ns	500 ns	200 ns	60 ns	100 ns	100 ns	60 ns
Program Energy	~ 45 pJ	~ 9 pJ	~ 420 fJ	~ 580 pJ	~ 500 pJ	~ 230 fJ	~ 1.2 nJ	~ 6 pJ	~ 100 fJ	~ 600 fJ	~ 3.4 pJ
Data Reten-tion	$> 10^2$ s	$> 10^5$ s	$> 10^4$ s	N.A.	$> 10^4$ s	$> 10^4$ s	$> 6\times10^5$ s	$> 10^4$ s	$> 10^4$ s	$> 10^4$ s	$> 10^4$ s
Memory States Per Layer of VRRAM	2	2	2	2	4	2	4	2	2	2	8

Table S7. The simulation results for 1T–1R, 1T–4R, and 1T–8R memory array *via* NVSim, with the transistor amount fixed at 8 MB, and different memory densities. We observe that by stacking more layers of VRRAMs, there is minimal change in the area, delay, and energy.

	Area (mm^2)	Read Delay (ns)	Write Delay (ns)	Read Energy (pJ)	Write Energy (nJ)
1T–1R	5.974	1.396	50.804	324.511	2.192
1T–4R	6.022	1.403	50.805	330.795	2.203
1T–8R	6.069	1.41	50.805	335.913	2.213

Table S8. Material properties for thermal simulation.

Material	Thermal Conductivity [W/(μm·K)]	Volumetric Heat Capacity [J/(μm^3·K)]
BEOL	2.25×10^{-6}	2.175×10^{-12}
Si	1.30×10^{-4}	2.20×10^{-12}
SiO_2	1.38×10^{-6}	1.62×10^{-12}
Pt	7.16×10^{-5}	2.90×10^{-12}
Au	3.17×10^{-4}	2.47×10^{-12}
HfO_x	1.38×10^{-6}	1.16×10^{-12}

Table S9. The layer structures for thermal simulation.

Layer	Material	Height (μm)
VRRAM	Pt	0.030
	HfO_x	0.005
	Pt	0.030
	SiO_2	0.100
Transistor	Au	0.040
	HfO_x	0.055
	Au	0.040
	SiO_2	0.100
PCB	BEOL Metal	10.00

Supplementary References

1 Zhao, W. & Cao, Y. New generation of predictive technology model for sub-45 nm early design exploration. *IEEE Trans. Electron Devices* **53**, 2816-2823 (2006).

2 Jiang, Z. *et al.* A compact model for metal–oxide resistive random access memory with experiment verification. *IEEE Trans. Electron Devices* **63**, 1884-1892 (2016).

3 Shulaker, M. M., Wu, T. F., Pal, A., Liang, Z. & Mitra, S. Monolithic 3D integration of logic and memory: Carbon nanotube FETs, resistive RAM, and silicon FETs. *2014 IEEE Int. Electron Devices Meeting (IEDM)* 27.4.1-27.4.4 (IEEE, 2014).

4 Moon, K., Kwak, M., Park, J., Lee, D. & Hwang, H. Improved conductance linearity and conductance ratio of 1T2R synapse device for neuromorphic systems. *IEEE Electron Device Lett* **38**, 1023-1026 (2017).

5 Shulaker, M. M. *et al.* Three-dimensional integration of nanotechnologies for computing and data storage on a single chip. *Nature* **547**, 74 (2017).

6 Wang, C.-H. *et al.* 3D monolithic stacked 1T1R cells using monolayer MoS_2 FET and hBN RRAM fabricated at low (150 °C) temperature. *2018 IEEE Int. Electron Devices Meeting (IEDM)* 22.5.1-22.5.4 (IEEE, 2018).

7 Wu, T. F. *et al.* Hyperdimensional computing exploiting carbon nanotube FETs, resistive RAM, and their monolithic 3D integration. *IEEE Journal of Solid-State Circuits* **53**, 3183-3196 (2018).

8 Hsieh, E. *et al.* High-density multiple bits-per-cell 1T4R RRAM array with gradual set/reset and its effectiveness for deep learning. *2019 IEEE Int. Electron Devices Meeting (IEDM)* 35.6.1-35.6.4 (IEEE,2019).

9 Sivan, M. *et al.* All WSe_2 1T1R resistive RAM cell for future monolithic 3D embedded memory integration. *Nat. Commun.* **10**, 1-12 (2019).

10 Su, C. *et al.* 3D integration of vertical-stacking of MoS_2 and Si CMOS featuring embedded 2T1R configuration demonstrated on full wafers. *2020 IEEE Int. Electron Devices Meeting (IEDM)* 12.2.1-12.2.4 (IEEE, 2020).

11 Li, Y. *et al.* Monolithic 3D integration of logic, memory and computing-in-memory for one-shot learning. *2021 IEEE Int. Electron Devices Meeting (IEDM)* 21.5.1-21.5.4 (IEEE, 2021).

12 Li, H. *et al.* Four-layer 3D vertical RRAM integrated with FinFET as a versatile computing unit for brain-inspired cognitive information processing. *2016 IEEE Symp. VLSI Technology* 1-2 (IEEE, 2016).

13 Hsieh, E. *et al*. Four-bits-per-memory one-transistor-and-eight-resistive-random-access-memory (1T8R) array. *IEEE Electron Device Lett.* **42**, 335-338 (2021).

14 Tang, B. *et al.* Wafer-scale solution-processed 2D material analog resistive memory

array for memory-based computing. *Nat. Commun.* **13**, 3037 (2022).

15 Chien, W. C. *et al.* Multi-layer sidewall WO_x resistive memory suitable for 3D ReRAM. *2012 IEEE Symp. VLSI Technology* 153-154 (IEEE, 2012).

16 Chen, H.-Y. *et al.* HfO_x based vertical resistive random access memory for cost-effective 3D cross-point architecture without cell selector. *2012 IEEE Int. Electron Devices Meeting (IEDM)* 20.7.1-20.2.4 (IEEE, 2012).

17 Hsu, C.-W. *et al.* 3D vertical TaO_x/TiO_2 RRAM with over 10^3 self-rectifying ratio and sub-μA operating current. *2013 IEEE Int. Electron Devices Meeting (IEDM)* 10.4.1-10.4.4 (IEEE, 2013).

18 Chen, H.-Y. *et al.* Experimental study of plane electrode thickness scaling for 3D vertical resistive random access memory. *Nanotechnology* **24**, 465201 (2013).

19 Bai, Y. *et al.* Study of multi-level characteristics for 3D vertical resistive switching memory. *Sci. Rep.* **4**, 1-7 (2014).

20 Lee, S., Sohn, J., Jiang, Z., Chen, H.-Y. & Philip Wong, H.-S. Metal oxide-resistive memory using graphene-edge electrodes. *Nat. Commun.* **6**, 1-7 (2015).

21 Yu, M. *et al.* Novel vertical 3D structure of TaO_x-based RRAM with self-localized switching region by sidewall electrode oxidation. *Sci. Rep.* **6**, 1-10 (2016).

22 Xu, X. *et al.* Fully CMOS compatible 3D vertical RRAM with self-aligned self-selective cell enabling sub-5nm scaling. *2016 IEEE Symp. VLSI Technology* 1-2 (IEEE, 2016).

23 Luo, Q., Xu, X., Gong, T., Lv, H. & Ming, L. 8-layers 3D vertical RRAM with excellent scalability towards storage class memory applications. *2017 IEEE Int. Electron Devices Meeting (IEDM)* 2.7.1-2.7.4 (IEEE, 2017).

24 Dong, X., Xu, C., Xie, Y. & Jouppi, N. P. Nvsim: A circuit-level performance, energy, and area model for emerging nonvolatile memory. *IEEE Trans. on Computer-Aided Design of Integrated Circuits and Systems* **31**, 994-1007 (2012).
